# Supplementary material for: American Board of Anesthesiology Mock Standardized Oral Examination Faculty Development Workshop
Source: MedEdPORTAL. 2021 Jul 29;17:11173. doi: 10.15766/mep_2374-8265.11173 (PMC8319152; doi:10.15766/mep_2374-8265.11173)
Supplement: Supplementary file 1 — Mock SOE Faculty Tip Sheet.pdfPart 1 Slide Presentation.pptxPart 2 Script, Stem, Questions & Evaluation.docxFacilitator Guide.docxFaculty Workshop Evaluation.docxFaculty Preintervention Survey.docxFaculty Postintervention Survey.docxResident Preintervention Survey.docxResident Postintervention Survey.docx [file mep_2374-8265.11173-s001.zip › A. Mock SOE Faculty Tip Sheet.pdf]

Mock Standardized Oral Exam (Mock-SOE) Preparation

Tip Sheet for Examiners

1. Setting the tone:

Greet the examinee as "Dr. (Last Name)" and introduce yourself in the same manner.
Let the examinee know they can refer to their notes.
Confirm that you have the same question stem.

2. Conducting the Exam:

Maintain a blank expression and neutral tone of voice, but remain pleasant. Maintain eye contact, while avoiding the use of cuing body language.

Avoid "teaching" during the exam. Don't ask follow-up questions to illustrate a teaching point, e.g. "well now the patient with the full stomach vomited during your standard induction" or "really, you'd do a spinal in a patient taking clopidogrel?!?" Stick to the script, as much as possible.

★ **Don't focus on content.** Try to keep to the script, but examples include

- Instead of asking "What PA pressure establishes the diagnosis of pulmonary hypertension?", Ask, "If the patient has pulmonary hypertension, how would it change your management?". If needed, follow up with "how" or "why/why not."
- Instead of asking "What is your cutoff for hyperkalemia?", ask "How would you lower the patient's potassium level", followed by "why did you choose that method?".
- Instead of asking "What is a normal pulse pressure variation", ask "What factors influence the accuracy of pulse pressure variation?".

Common Candidate Problems	Suggested Solutions
**Candidate gives "yes" or "no" answers**	Prompt them with "why" or "why not".
**Candidate giving overly lengthy answers**	Gently interrupt.
**Candidate isn't choosing an answer**	Restate question in a different manner once, then move to another question and provide feedback at the end of the examination.
**Candidate asks a question**	Reply with "how would that change your management." No need to fabricate data. Probe their thought process on why they want that additional information.
**Candidate can't answer or is melting down**	Let them off the hook! "Let's move on…" Feel free to offer a brief reassurance instruction, such as "take a deep breath; you're welcome to have a drink of water. Let's move on to a different question."

3. Feedback:

Feedback should be about the attributes of a successful candidate and not clinical content.

Attribute	Meaning	How to Assess
**Judgment**	*What is the candidate's overall plan? Are their decisions sound?*	Pay attention to the candidate's overall anesthetic plan and decision-making about overall case.
**Adaptability**	*How well does the candidate deal with a change?*	Add something new. "The patient refuses a block, now what do you do?"
**Application of Knowledge**	*Does the candidate demonstrate that their fund of knowledge behind their answers?*	When the candidate answers your "why" questions, do they answer from a clear fund of knowledge?
**Organization**	*Does the candidate jump around or do they follow a logical order in their presentation of information?*	Pay attention to candidate's ordering of information. Can you follow the answer easily or are you confused?
