# Supplementary material for: American Board of Anesthesiology Mock Standardized Oral Examination Faculty Development Workshop
Source: MedEdPORTAL. 2021 Jul 29;17:11173. doi: 10.15766/mep_2374-8265.11173 (PMC8319152; doi:10.15766/mep_2374-8265.11173)
Supplement: Supplementary file 1 — Mock SOE Faculty Tip Sheet.pdfPart 1 Slide Presentation.pptxPart 2 Script, Stem, Questions & Evaluation.docxFacilitator Guide.docxFaculty Workshop Evaluation.docxFaculty Preintervention Survey.docxFaculty Postintervention Survey.docxResident Preintervention Survey.docxResident Postintervention Survey.docx [file mep_2374-8265.11173-s001.zip › B. Part 1 Slide Presentation.pptx]

## Slide 1
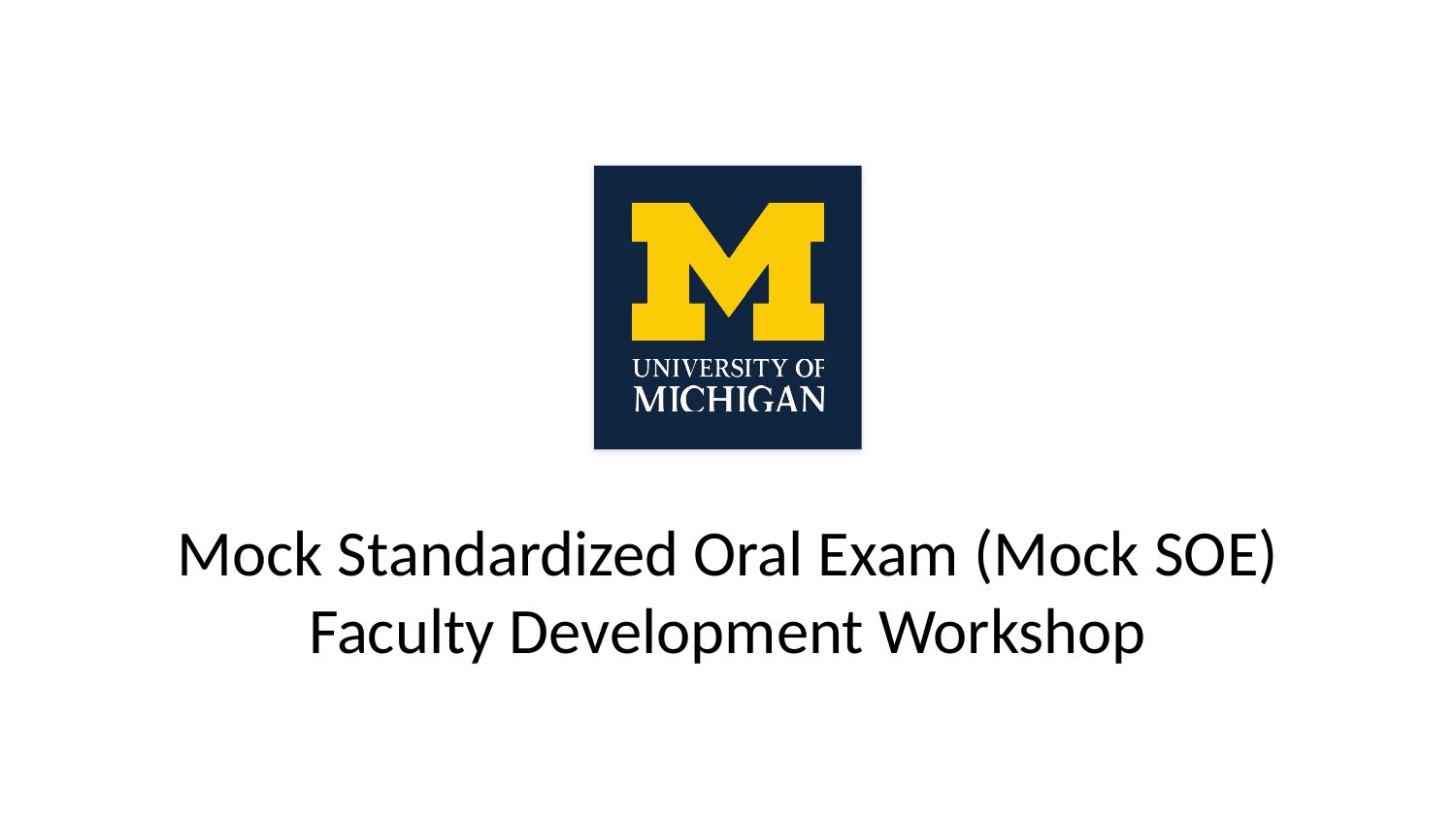

Mock Standardized Oral Exam (Mock SOE)
Faculty Development Workshop

## Slide 2
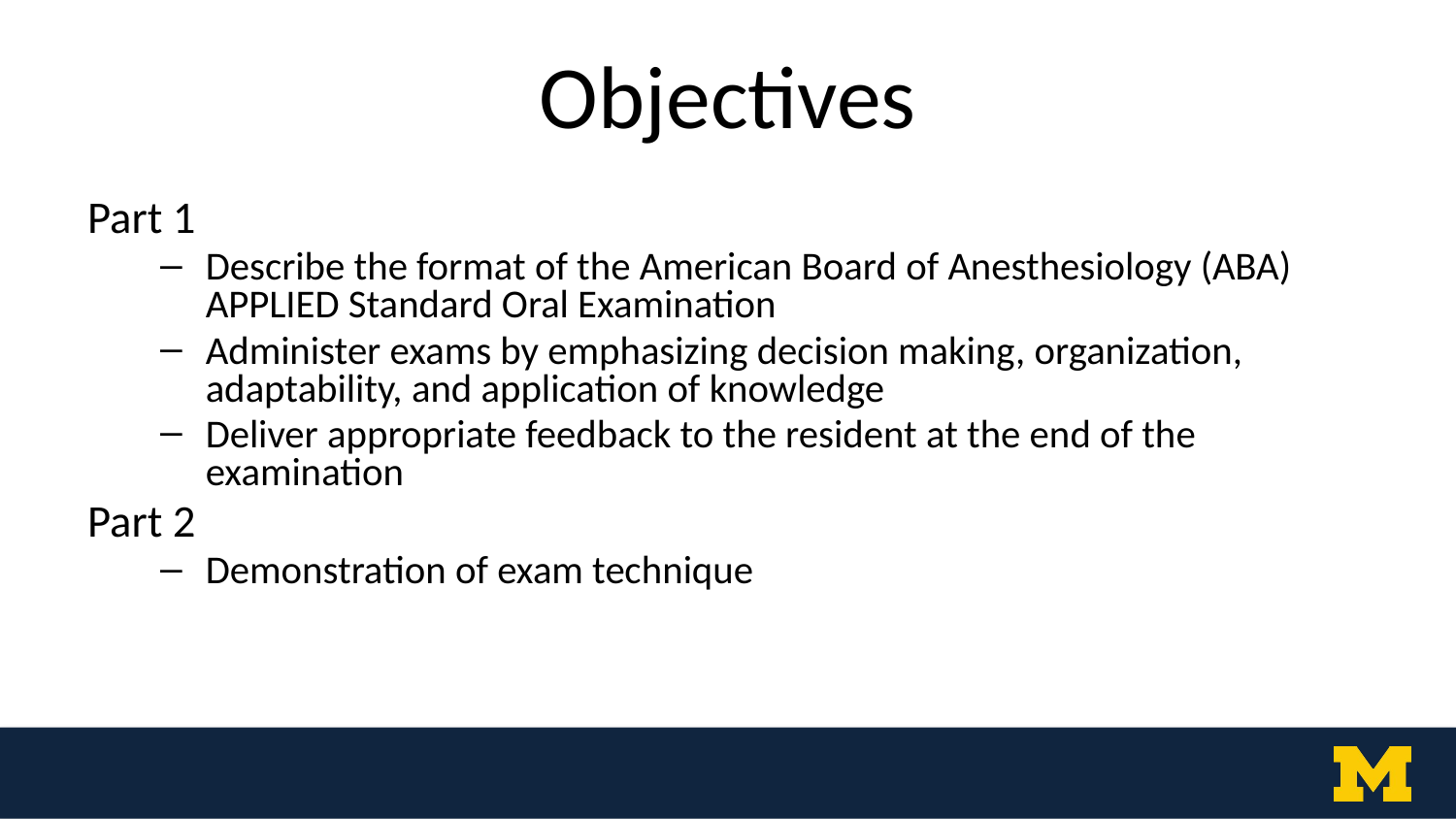

# Objectives
Part 1
Describe the format of the American Board of Anesthesiology (ABA) APPLIED Standard Oral Examination
Administer exams by emphasizing decision making, organization, adaptability, and application of knowledge
Deliver appropriate feedback to the resident at the end of the examination
Part 2
Demonstration of exam technique

## Slide 3
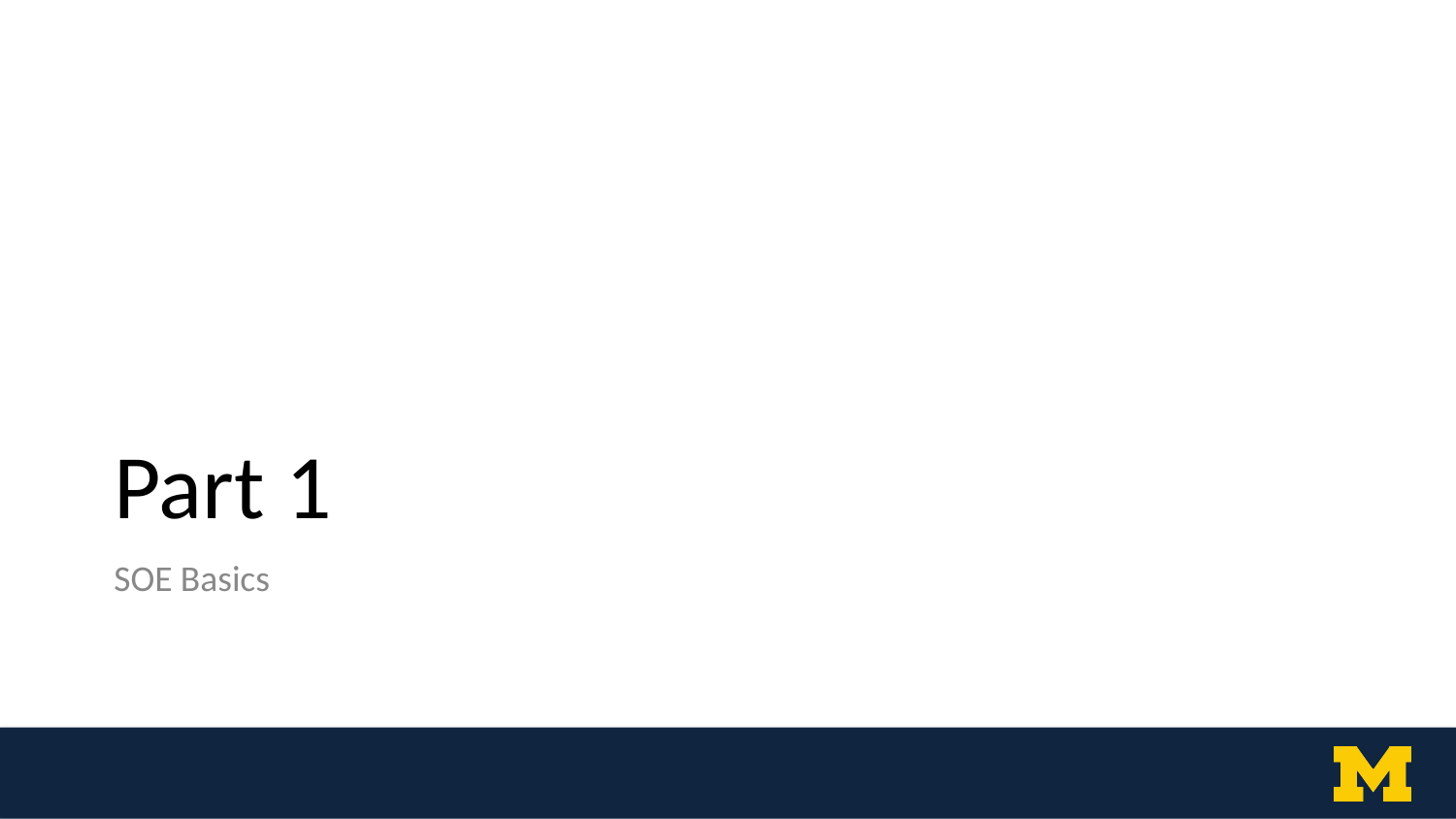

# Part 1
SOE Basics

## Slide 4
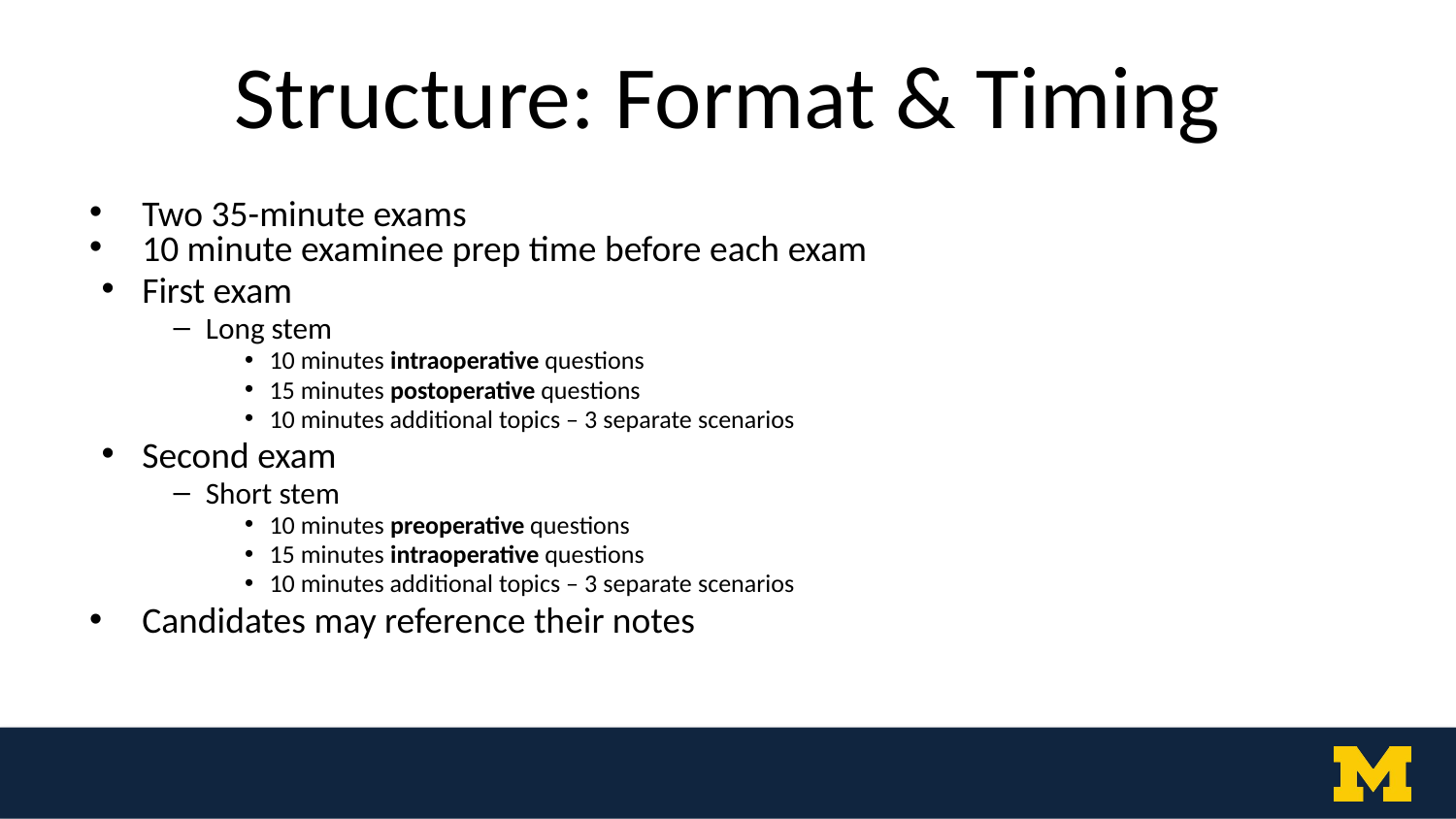

# Structure: Format & Timing
Two 35-minute exams
10 minute examinee prep time before each exam
First exam
Long stem
10 minutes intraoperative questions
15 minutes postoperative questions
10 minutes additional topics – 3 separate scenarios
Second exam
Short stem
10 minutes preoperative questions
15 minutes intraoperative questions
10 minutes additional topics – 3 separate scenarios
Candidates may reference their notes

## Slide 5
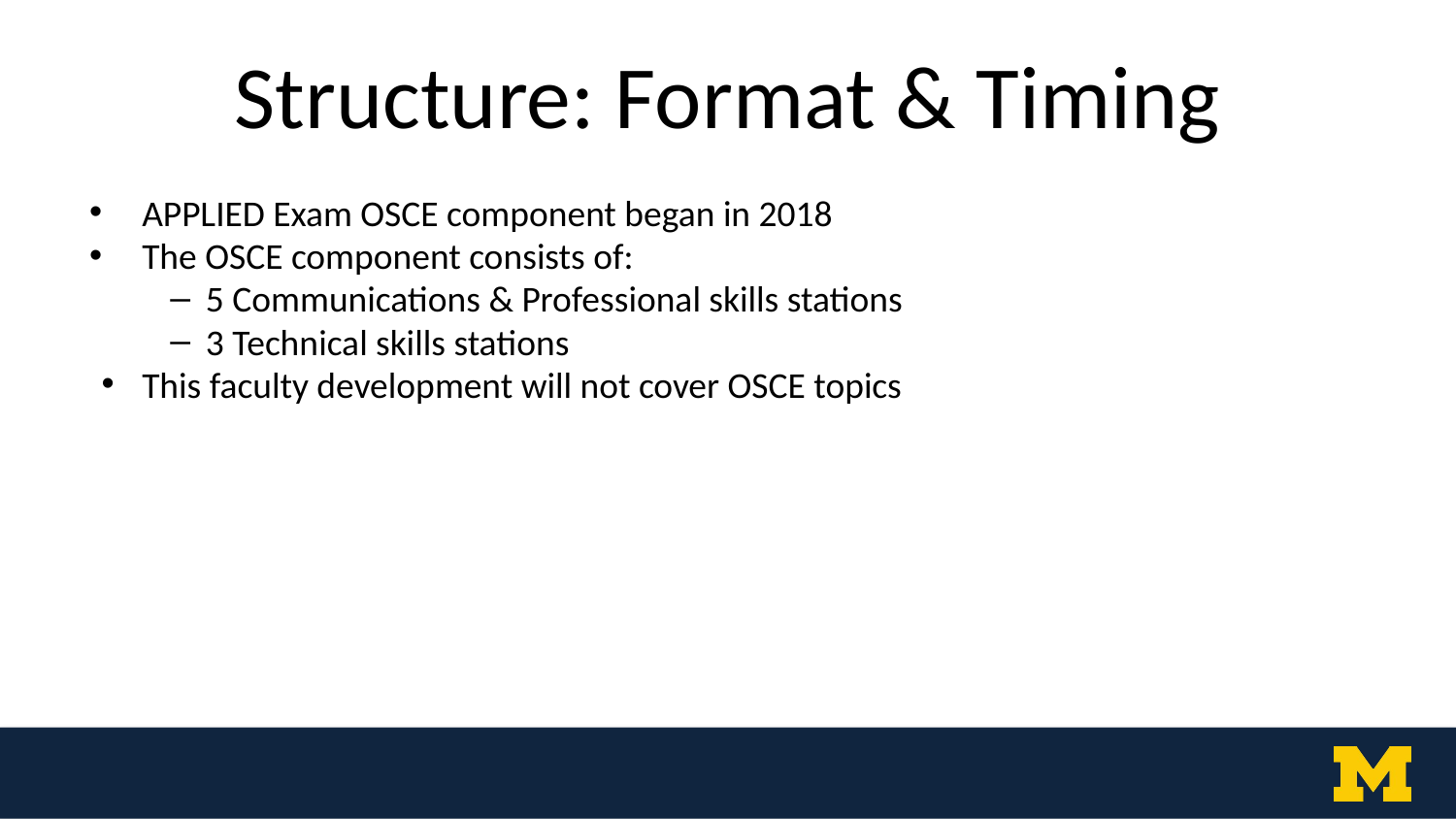

# Structure: Format & Timing
APPLIED Exam OSCE component began in 2018
The OSCE component consists of:
5 Communications & Professional skills stations
3 Technical skills stations
This faculty development will not cover OSCE topics

## Slide 6
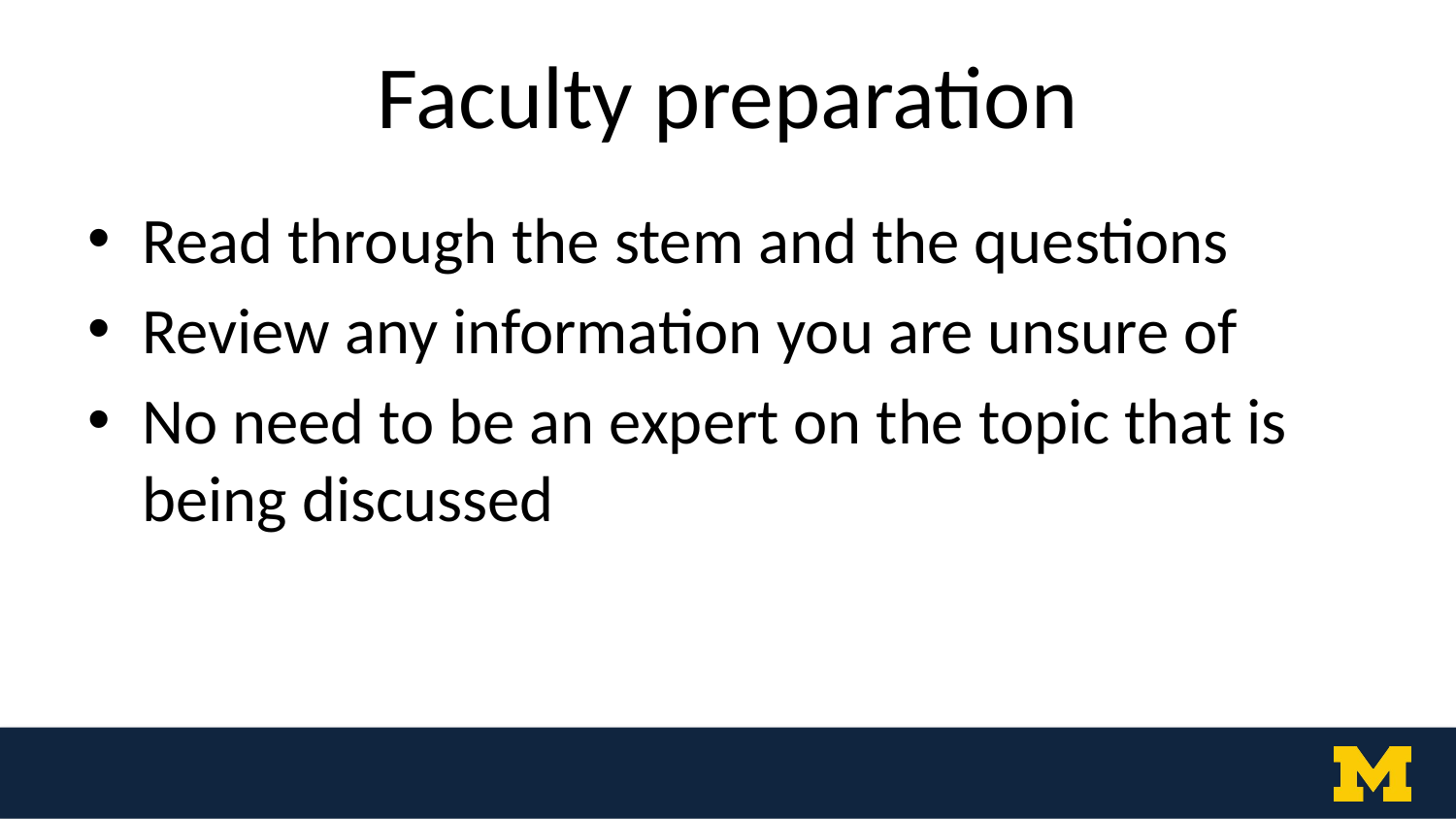

# Faculty preparation
Read through the stem and the questions
Review any information you are unsure of
No need to be an expert on the topic that is being discussed

## Slide 7
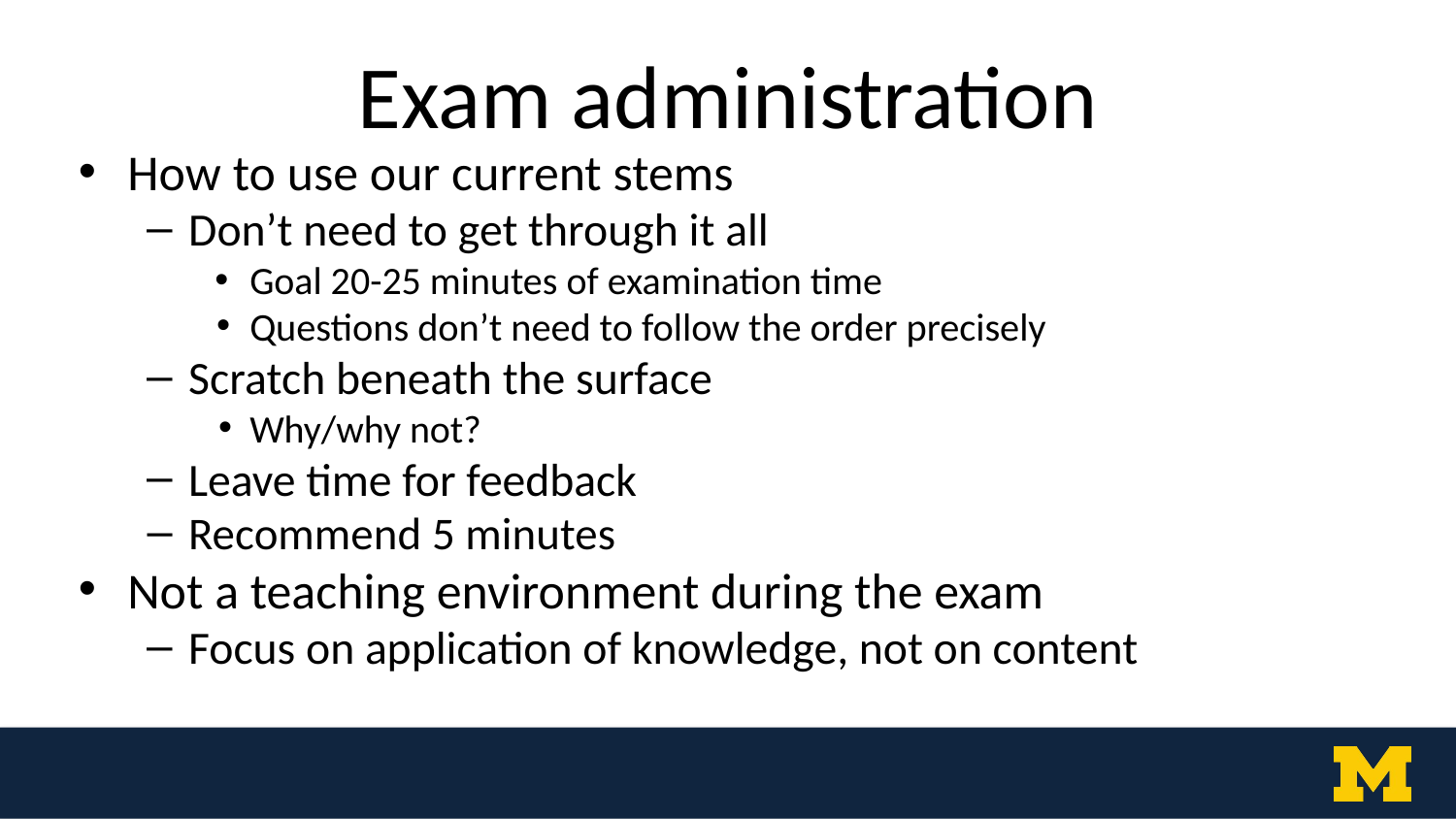

# Exam administration
How to use our current stems
Don’t need to get through it all
Goal 20-25 minutes of examination time
Questions don’t need to follow the order precisely
Scratch beneath the surface
Why/why not?
Leave time for feedback
Recommend 5 minutes
Not a teaching environment during the exam
Focus on application of knowledge, not on content

## Slide 8
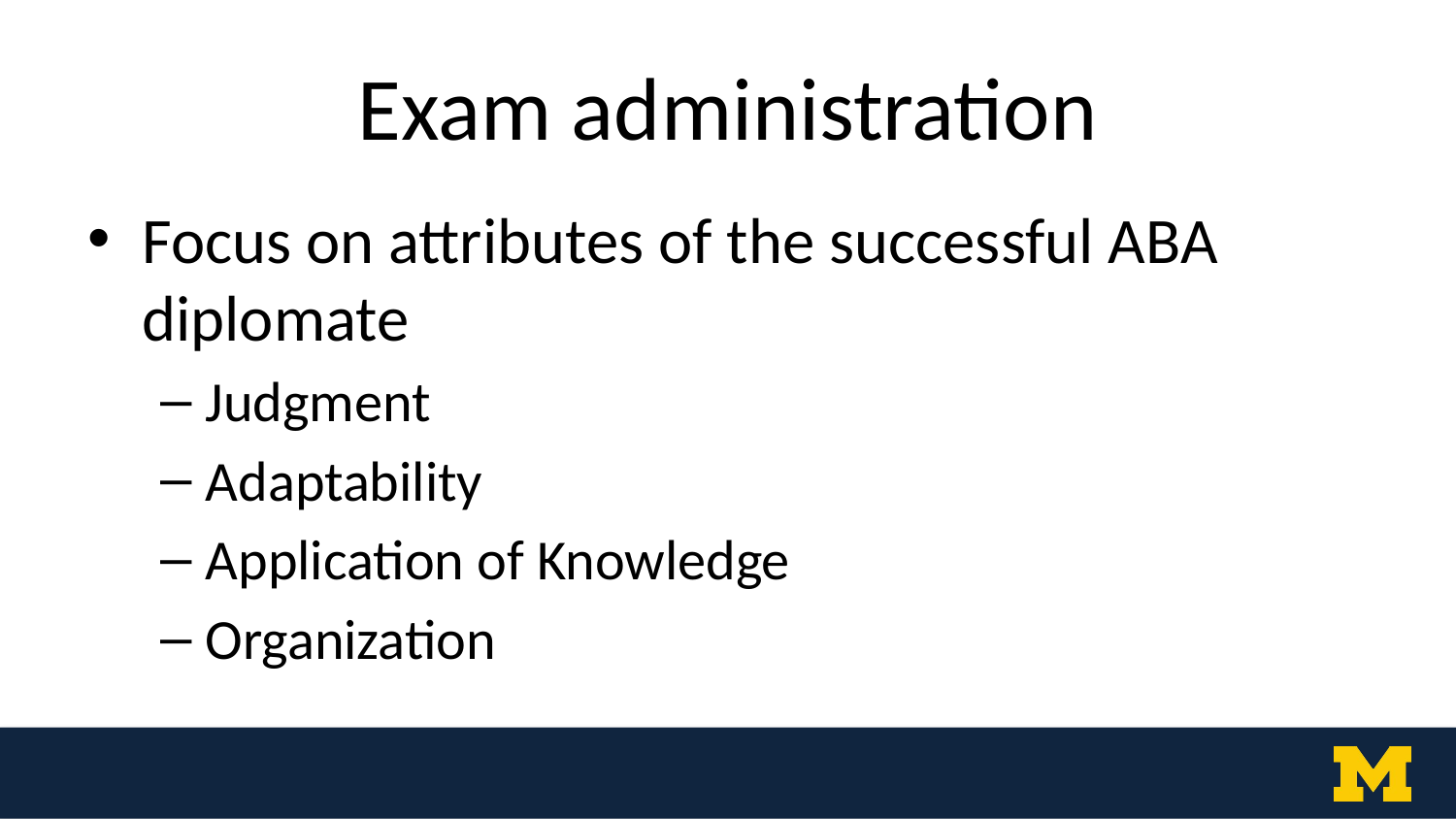

# Exam administration
Focus on attributes of the successful ABA diplomate
Judgment
Adaptability
Application of Knowledge
Organization

## Slide 9
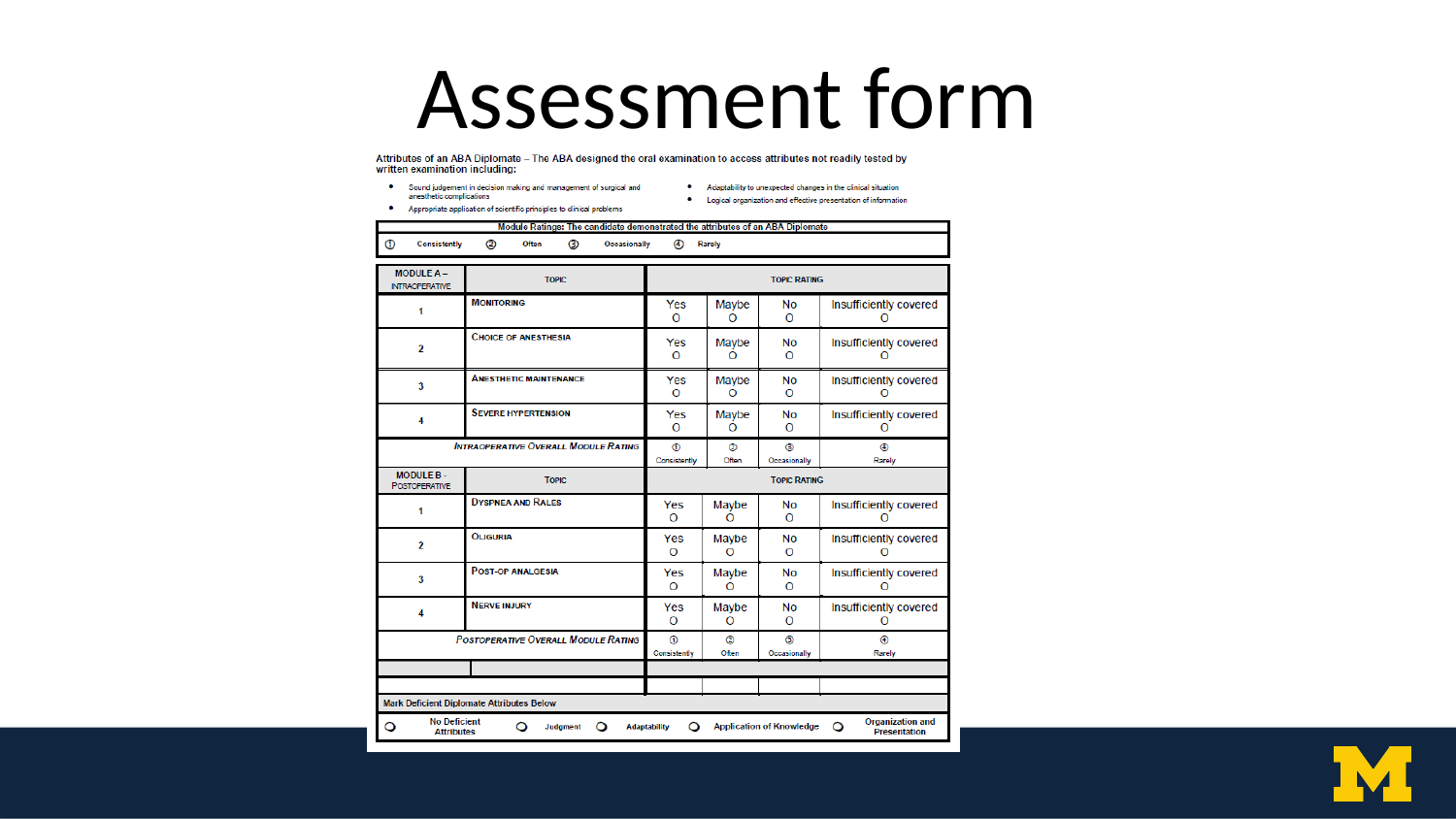

# Assessment form

## Slide 10
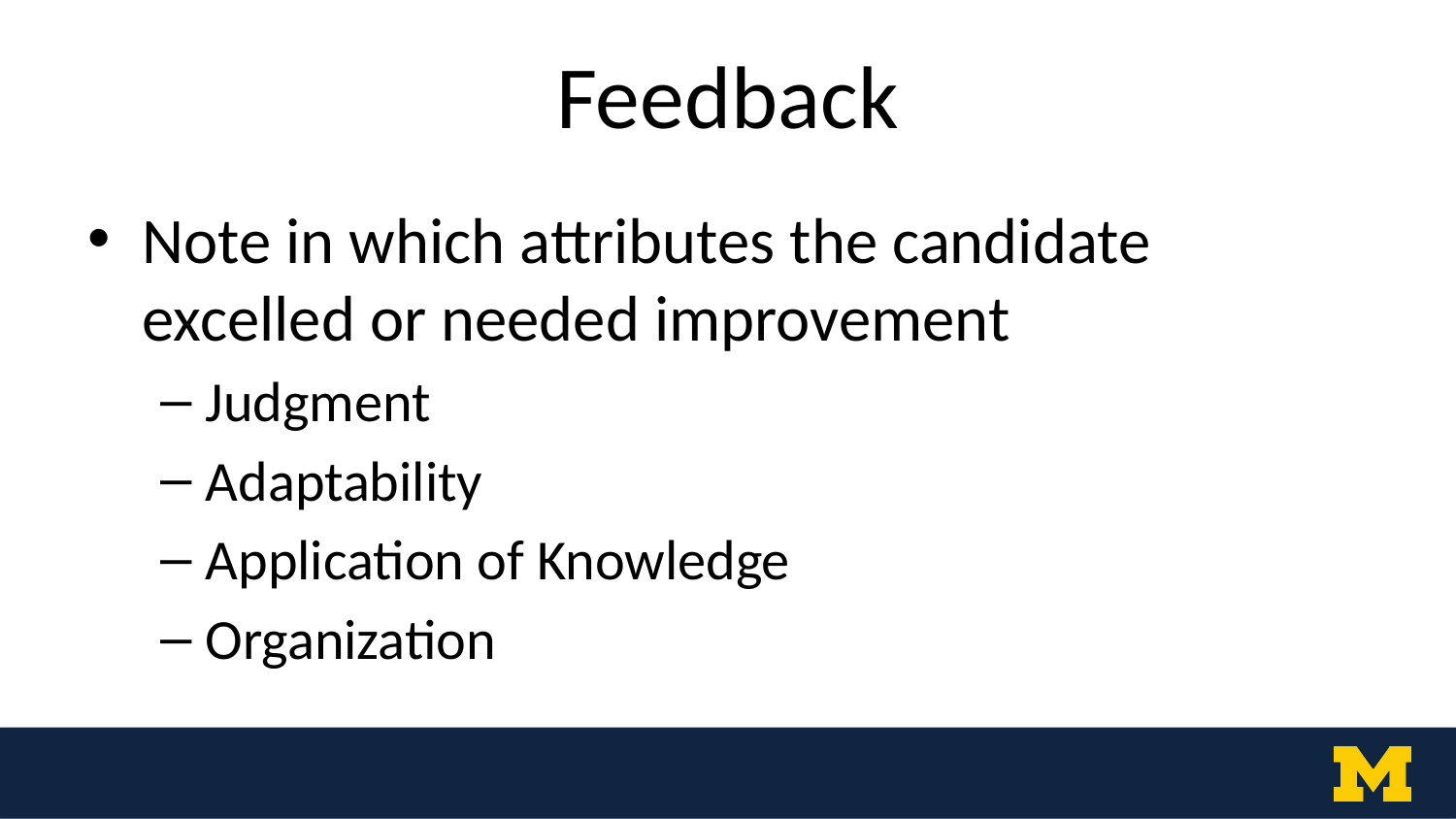

# Feedback
Note in which attributes the candidate excelled or needed improvement
Judgment
Adaptability
Application of Knowledge
Organization

## Slide 11
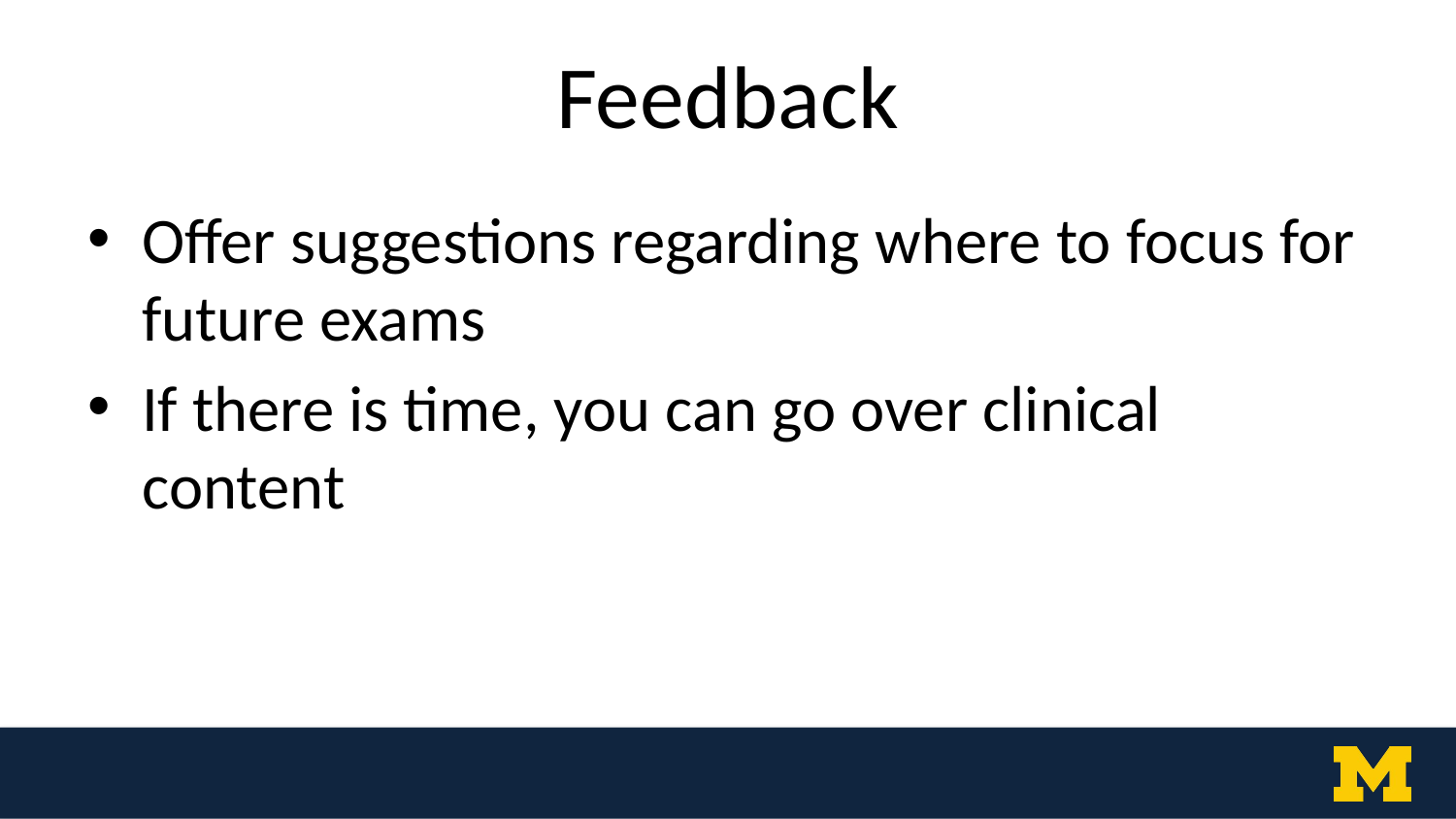

# Feedback
Offer suggestions regarding where to focus for future exams
If there is time, you can go over clinical content

## Slide 12
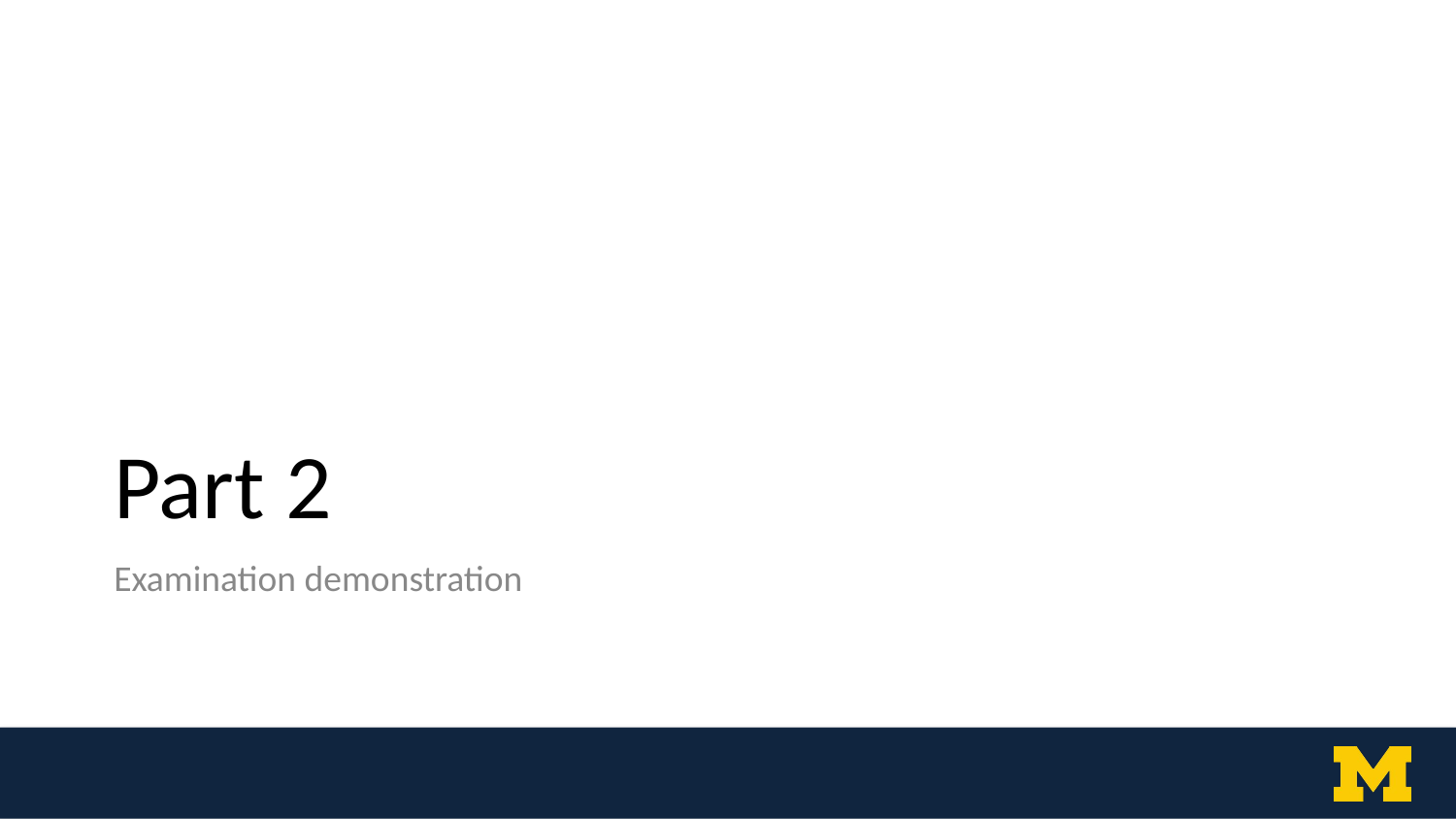

# Part 2
Examination demonstration

## Slide 13
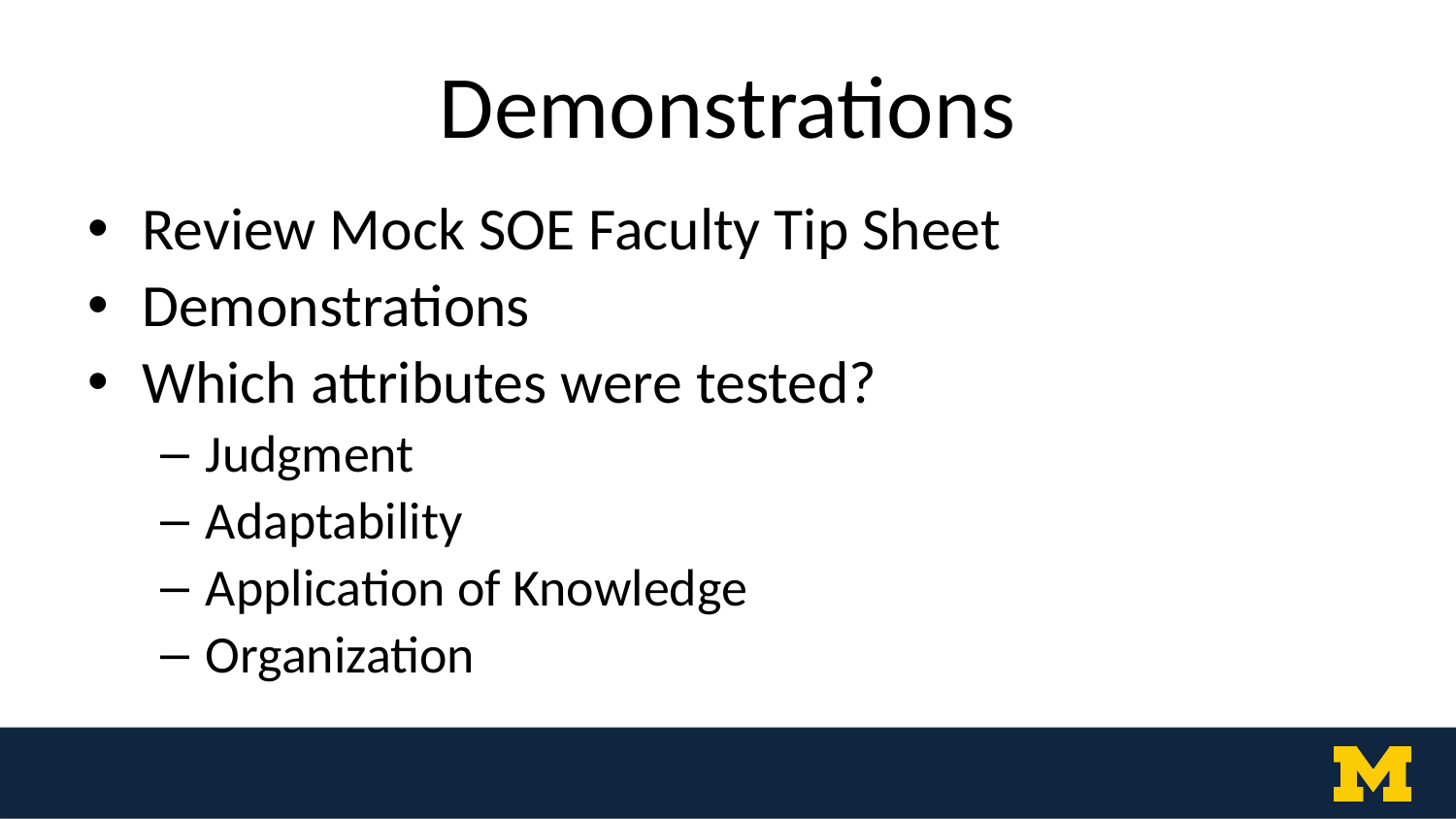

# Demonstrations
Review Mock SOE Faculty Tip Sheet
Demonstrations
Which attributes were tested?
Judgment
Adaptability
Application of Knowledge
Organization

## Slide 14
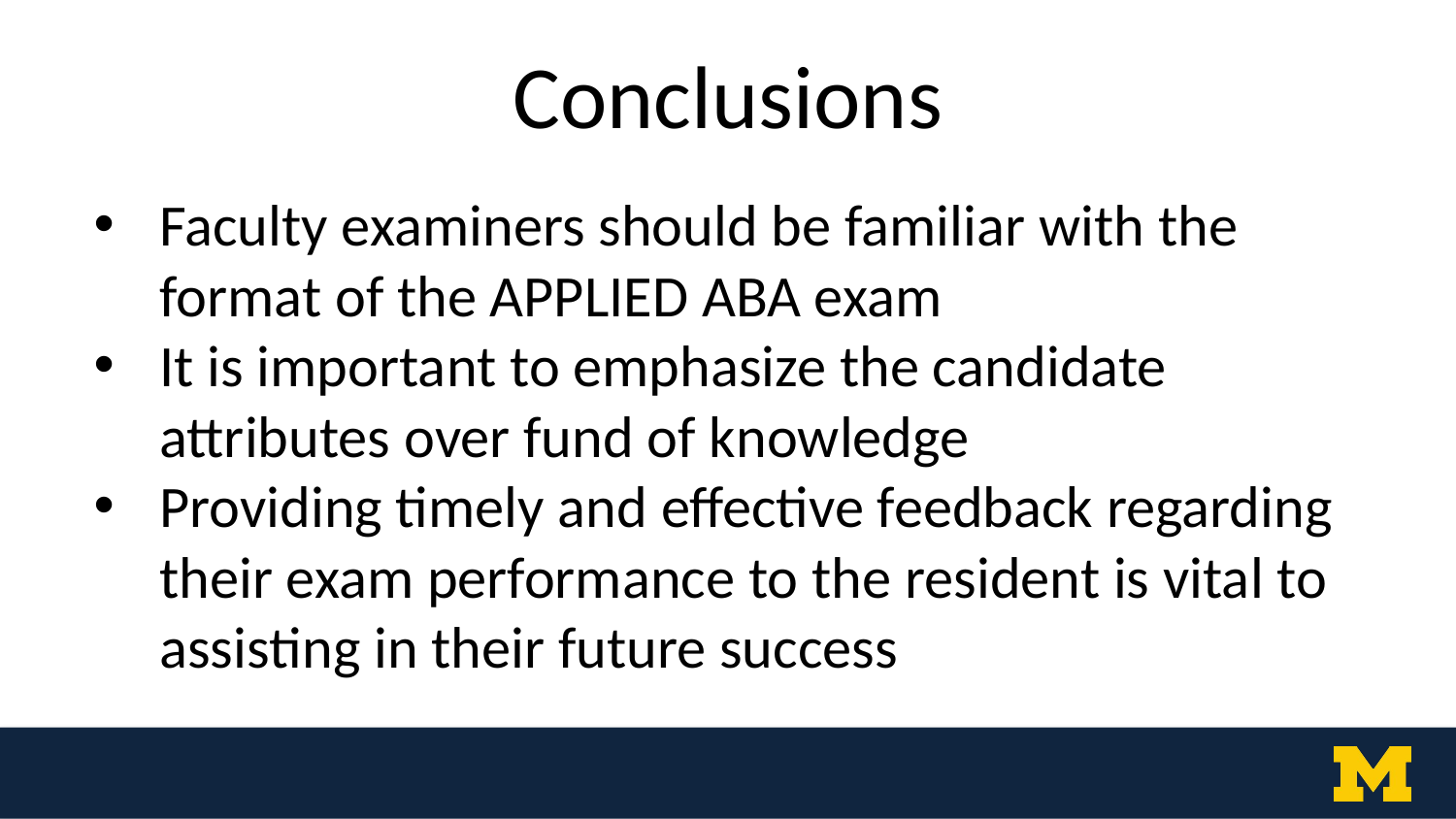

# Conclusions
Faculty examiners should be familiar with the format of the APPLIED ABA exam
It is important to emphasize the candidate attributes over fund of knowledge
Providing timely and effective feedback regarding their exam performance to the resident is vital to assisting in their future success

## Slide 15
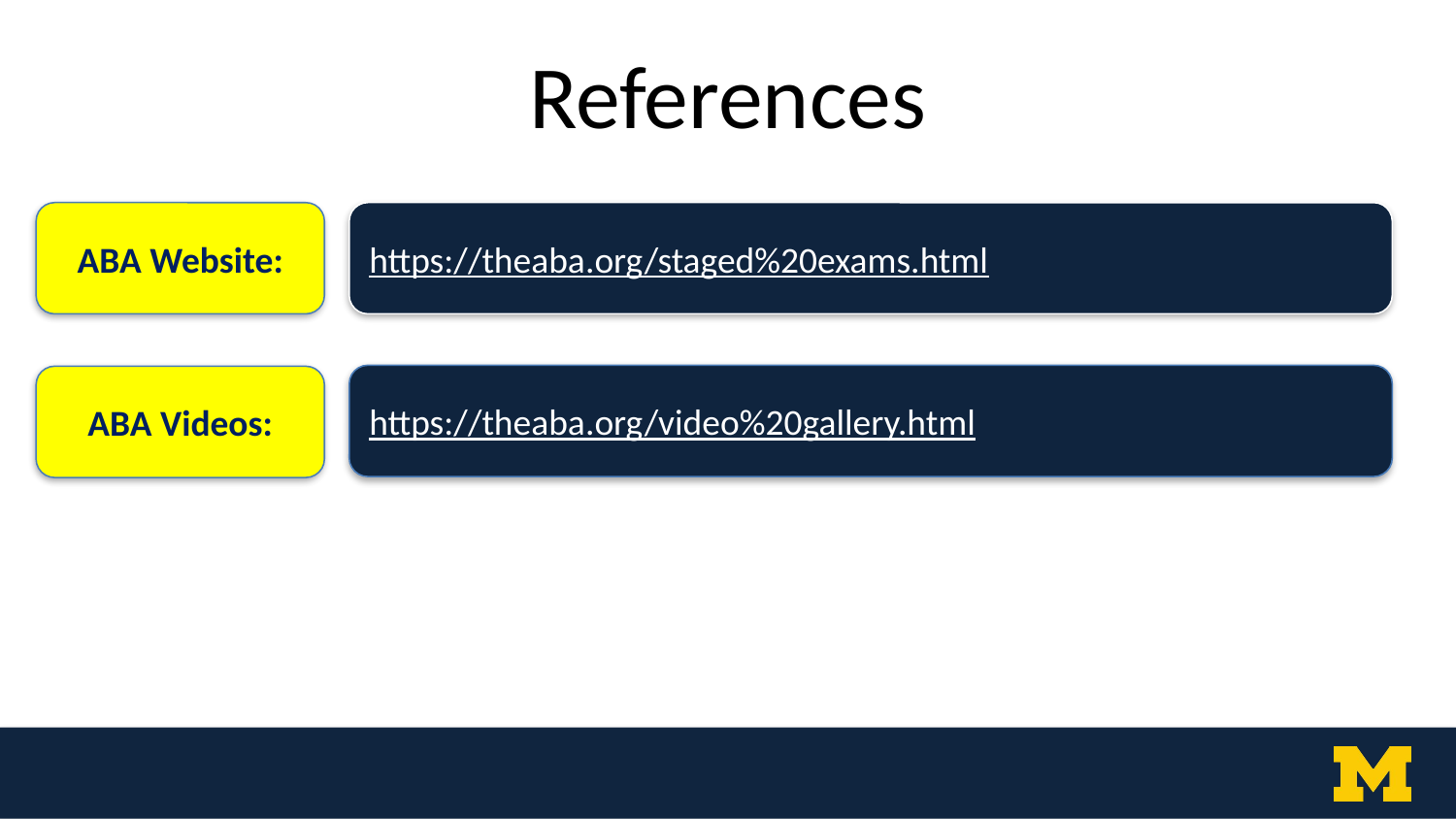

# References
ABA Website:
https://theaba.org/staged%20exams.html
https://theaba.org/video%20gallery.html
ABA Videos:

## Slide 16
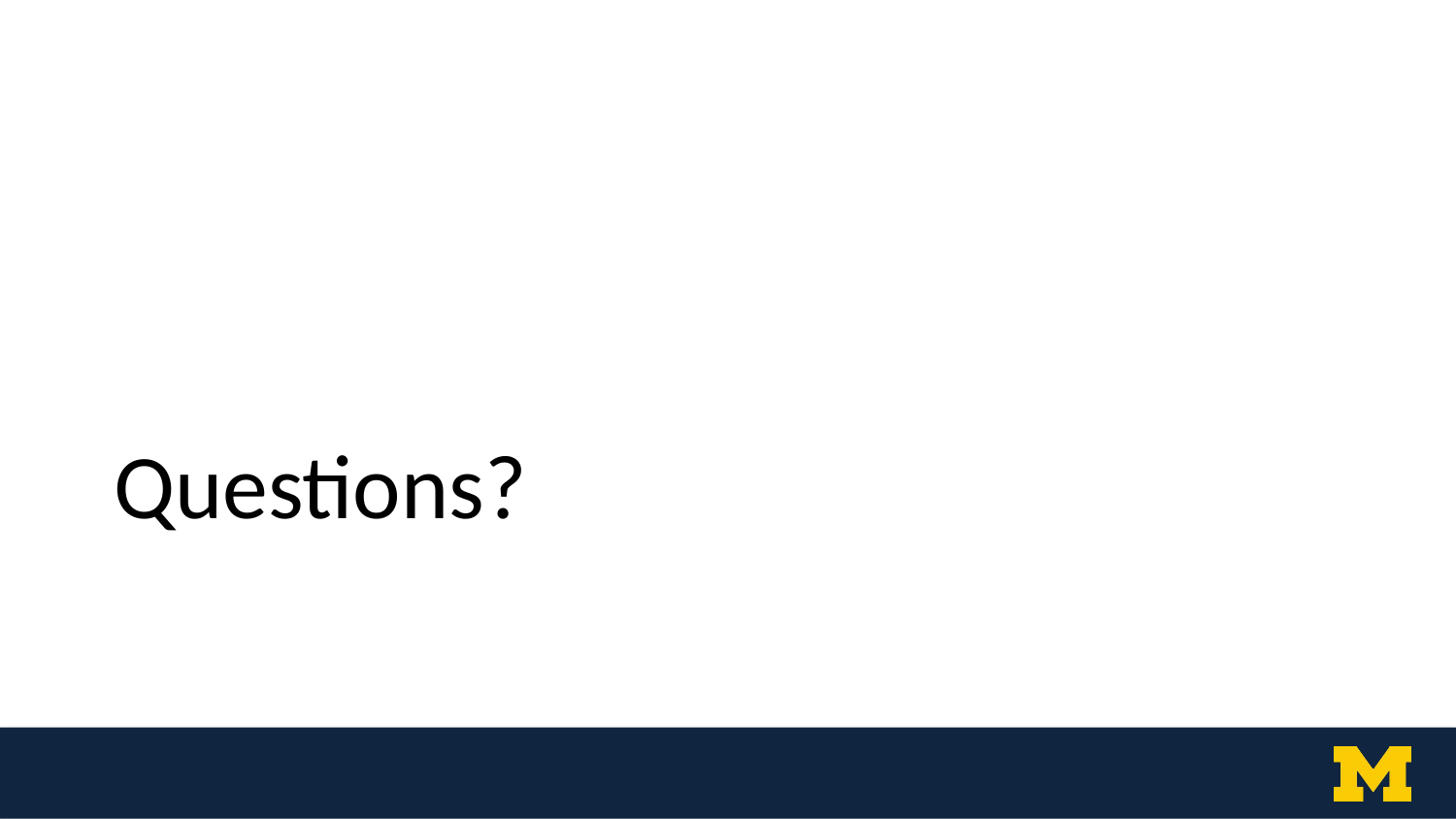

# Questions?
