# Supplementary material for: American Board of Anesthesiology Mock Standardized Oral Examination Faculty Development Workshop
Source: MedEdPORTAL. 2021 Jul 29;17:11173. doi: 10.15766/mep_2374-8265.11173 (PMC8319152; doi:10.15766/mep_2374-8265.11173)
Supplement: Supplementary file 1 — Mock SOE Faculty Tip Sheet.pdfPart 1 Slide Presentation.pptxPart 2 Script, Stem, Questions & Evaluation.docxFacilitator Guide.docxFaculty Workshop Evaluation.docxFaculty Preintervention Survey.docxFaculty Postintervention Survey.docxResident Preintervention Survey.docxResident Postintervention Survey.docx [file mep_2374-8265.11173-s001.zip › C. Part 2 Script, Stem, Questions & Evaluation.docx]

A 50-year-old 58 kg woman is scheduled for a kidney transplant. The organ was procured from a cadaveric donor 16 hours ago.

HPI: Chronic renal failure secondary to hypertension

Hemodialysis three times weekly for 8 years; last dialysis was 36 hours

ago

Hypertension for 24 years with an episode of acute pulmonary edema one year ago (heart failure with preserved ejection fraction; HFpEF)

MEDS: Nifedipine, lisinopril, ranitidine

PMH: Symptomatic hiatal hernia

Allergy to PCN (hives)

EXAM: T 37° C; BP 175/110 mmHg; HR 95; R 20; SpO2 96%

Normal airway exam

Lungs clear to auscultation

Heart sounds normal

AV fistula present in the left forearm

CXR: Heart size normal

No pleural effusion

Prominent vascular markings

ECG: LVH with nonspecific ST-T wave changes

Echo: Left ventricular concentric hypertrophy

No inducible ischemia on dobutamine stress

Diastolic dysfunction noted

LABS: Hgb 8.0 gm/dL; Na 135 mEq/L; K 5.6 mEq/L; BUN 49 mg/dL; Creatinine 5.0 mg/dL

On arrival to the preoperative holding room, a 20 gauge IV catheter is present in the right dorsal hand. The patient is anxious.

The questions for the long stem in this demonstration are taken from the intraoperative management module. There are suggested examiner questions and examinee responses for those playing these roles.

This section will focus on asking questions which will allow for assessment of the examinee’s judgment and adaptability

**Choice of anesthesia:**

Examiner:

Are you considering placing an epidural for this patient? What are the potential benefits of regional anesthesia in this patient for this operation?

Would you perform an epidural anesthetic as the sole technique for operative anesthesia? Why/why not?

Examinee:

Should answer questions as if they would consider offering an epidural to this patient in order for intraoperative and postoperative analgesia, but would also perform a general anesthetic.

What local anesthetic solution would you select? How would the presence of this patient’s renal failure affect your choice local anesthetic?

Examiner:

The patient refuses to have an epidural placed. Would you alter your anesthetic plan?

Examiner:

What are your goals for induction of anesthesia in this patient? How will you secure the airway? What if this patient reports to you a history of a difficult intubation at a different institution?

This section will focus on asking questions which will allow for assessment of the examinee’s application of knowledge.

**Anesthetic maintenance:**

Examiner:

What is your plan for anesthetic maintenance? Would a total intravenous anesthetic technique be appropriate for this case? Why/why not? If you were to proceed with TIVA, what infusions would you want to use? Is propofol appropriate? Why/why not? Would remifentanil be appropriate? Why/why not? Would you avoid nitrous oxide? Why/why not?

The examiner can also ask more technical questions to serve as an example of asking inappropriate knowledge questions. Such as:

What receptors does propofol act at? How is remifentanil metabolized?

This section will focus on asking questions which will allow for assessment of the examinee’s organization.

**Severe hypertension:**

Examiner:

Upon surgical incision, the BP increases to 240/140 mmHg. How would you manage this? Why is it important to treat hypertension of this severity?

Examinee:

The examinee can choose to answer in an organized or disorganized manner.

Examiner:

If you saw concomitant ST segments changes with the hypertension, is it reasonable to deepen the inhalation anesthetic? What drugs would you give to control the hypertension?

Examinee:

The examinee can back track to the previous question in order to continue showing a disorganized thought process

# Attributes of an ABA Diplomate – The ABA designed the oral examination to access attributes not readily tested by written examination including:

| - Sound judgment in decision making and management of surgical and anesthetic complications - Appropriate application of scientific principles to clinical problems | - Adaptability to unexpected changes in the clinical situation - Logical organization and effective presentation of information |
| --- | --- |

| **Module Ratings: The candidate demonstrated the attributes of an ABA Diplomate** | | | | | | | |
| --- | --- | --- | --- | --- | --- | --- | --- |
| **①** | **Consistently** | **②** | **Often** | **③** | **Occasionally** | **④** | **Rarely** |

| **MODULE A –**  intraoperative | | **Topic** | | | | | | **Topic RatinG** | | | | | | | |
| --- | --- | --- | --- | --- | --- | --- | --- | --- | --- | --- | --- | --- | --- | --- | --- |
| **1** | | **Monitoring** | | | | | | Yes  O | | | Maybe  O | No  O | Insufficiently covered  O | | |
| **2** | | **Choice of anesthesia** | | | | | | Yes  O | | | Maybe  O | No  O | Insufficiently covered  O | | |
| **3** | | **Anesthetic maintenance** | | | | | | Yes  O | | | Maybe  O | No  O | Insufficiently covered  O | | |
| **4** | | **Severe hypertension** | | | | | | Yes  O | | | Maybe  O | No  O | Insufficiently covered  O | | |
| ***Intraoperative Overall Module Rating*** | | | | | | | | ①  Consistently | | | ②  Often | ③  Occasionally | ④  Rarely | | |
| **MODULE B -** Postoperative | | **Topic** | | | | | | **Topic Rating** | | | | | | | |
| **1** | | **Dyspnea and Rales** | | | | | | Yes  O | | Maybe  O | | No  O | Insufficiently covered  O | | |
| **2** | | **Oliguria** | | | | | | Yes  O | | Maybe  O | | No  O | Insufficiently covered  O | | |
| **3** | | **Post-op analgesia** | | | | | | Yes  O | | Maybe  O | | No  O | Insufficiently covered  O | | |
| **4** | | **Nerve injury** | | | | | | Yes  O | | Maybe  O | | No  O | Insufficiently covered  O | | |
| ***Postoperative Overall Module Rating*** | | | | | | | | ①  Consistently | | ②  Often | | ③  Occasionally | ④  Rarely | | |
|  | | |  | | | | |  | | | | | | | |
|  | | | | | | | |  | |  | |  |  | | |
| **Mark Deficient Diplomate Attributes Below** | | | | | | | | | | | | | | | |
| **🔾** | **No Deficient Attributes** | | | **🔾** | **Judgment** | **🔾** | **Adaptability** | | **🔾** | | **Application of Knowledge** | | | **🔾** | **Organization and Presentation** |
