# Supplementary material for: American Board of Anesthesiology Mock Standardized Oral Examination Faculty Development Workshop
Source: MedEdPORTAL. 2021 Jul 29;17:11173. doi: 10.15766/mep_2374-8265.11173 (PMC8319152; doi:10.15766/mep_2374-8265.11173)
Supplement: Supplementary file 1 — Mock SOE Faculty Tip Sheet.pdfPart 1 Slide Presentation.pptxPart 2 Script, Stem, Questions & Evaluation.docxFacilitator Guide.docxFaculty Workshop Evaluation.docxFaculty Preintervention Survey.docxFaculty Postintervention Survey.docxResident Preintervention Survey.docxResident Postintervention Survey.docx [file mep_2374-8265.11173-s001.zip › D. Facilitator Guide.docx]

**Facilitator’s Guide**

This session is intended to be a faculty development workshop for anesthesiology faculty at academic institutions responsible for preparing anesthesiology trainees for the American Board of Anesthesiology (ABA) APPLIED Standard Oral Examination(SOE).

One or two faculty should be the facilitators. Two faculty are recommended for the interactive Part 2. It is not necessary for the faculty facilitator to be ABA Examiners. To assist with preparation, it is advised that faculty facilitators review the information regarding the ABA APPLIED exam on the ABA website. [www.theaba.org](http://www.theaba.org). There are videos as well on the ABA website that provide an overview of the exam administration. The information provided by the ABA in conjunction with this *MedEdPORTAL* publication and personal experience should provide faculty with sufficient information to guide this session.

The optimal setup for this presentation is in a conference room where there is access to a computer, projection screen and room for faculty to work in pairs. The overall session should last approximately 60 minutes.

*Guide to Part 1*

Part 1 is a didactic power point presentation. The slides are included as Appendix B. The presentation can be given by one or two faculty.

The length of the presentation should be about 30 minutes.

This part will cover the standard format of the SOE, the candidate attributes the ABA aims to assess, and techniques for providing feedback. Notes are included for the slides.

Some of the information can be adapted to accommodate how the individual training program conducts their Mock SOEs.

Allow time for questions.

*Guide to Part 2*

Part 2 is a demonstration by the 2 faculty facilitators and should take about 30 minutes.

One faculty moderator will play the role of the examiner, the other the role of candidate/resident.

A stem and questions are provided for you in Appendix C. You can also have copies of the stem printed to provide for participants. The script and questions may be modified as the facilitators feel is appropriate.

The Mock SOE Faculty Tip Sheet (Appendix A) should also be distributed to the workshop participants.

It is suggested to stop at the end of each highlighted section during this interactive part to allow for discussion among learners.

It is helpful for the participants to all see this demonstration. If there are more than 2 facilitators with a larger number of participants, the larger group can break up into smaller groups.

The authors elected not to include a recording of this demonstration to allow for a more individualized approach and time for questions. It is certainly possible that facilitators could record their demonstration to be able to use at future sessions.

*Evaluations*

Included in this submission are multiple evaluations.

Appendix E is an evaluation of the workshop itself.

Appendix F is an evaluation for faculty examiners to complete prior to attending the session or receiving the Mock SOE Faculty Tip sheet (Appendix A).

Appendix G is an evaluation for faculty examiners to complete after the intervention and administering a Mock SOE.

Appendix H is an evaluation for residents to complete prior to the intervention occurring at your institution. Appendix I is then an evaluation for residents to complete following participation in a Mock SOE after the intervention.
