# Supplementary material for: American Board of Anesthesiology Mock Standardized Oral Examination Faculty Development Workshop
Source: MedEdPORTAL. 2021 Jul 29;17:11173. doi: 10.15766/mep_2374-8265.11173 (PMC8319152; doi:10.15766/mep_2374-8265.11173)
Supplement: Supplementary file 1 — Mock SOE Faculty Tip Sheet.pdfPart 1 Slide Presentation.pptxPart 2 Script, Stem, Questions & Evaluation.docxFacilitator Guide.docxFaculty Workshop Evaluation.docxFaculty Preintervention Survey.docxFaculty Postintervention Survey.docxResident Preintervention Survey.docxResident Postintervention Survey.docx [file mep_2374-8265.11173-s001.zip › E. Faculty Workshop Evaluation.docx]

**Mock Standardized Oral Examination(SOE) Faculty Development Session Evaluation**

Q1 Were you satisfied with the workshop?

- Extremely satisfied
- Somewhat satisfied
- Neither satisfied nor dissatisfied
- Somewhat dissatisfied
- Extremely dissatisfied

Q2 Do you feel the following objectives were met?

|  | Fully Met | Partially Met | Not Met |
| --- | --- | --- | --- |
| Describe the format of the American Board of Anesthesiology (ABA) Applied Standard Oral Examination |  |  |  |
| Administer exams by emphasizing decision making, organization, adaptability, and application of knowledge |  |  |  |
| Deliver appropriate feedback to the resident at the end of the examination |  |  |  |

Q3 As a result of participating in the session, will you try to make any changes to the way you deliver mock SOEs?

- Yes
- Maybe
- No

Q4 If yes, please list one specific change you might make in practice.

________________________________________________________________

________________________________________________________________

Q5 If no, why not?

________________________________________________________________

________________________________________________________________

Q6 Let us know what you think. What went well? What could we have done better?

________________________________________________________________

________________________________________________________________
