# Supplementary material for: American Board of Anesthesiology Mock Standardized Oral Examination Faculty Development Workshop
Source: MedEdPORTAL. 2021 Jul 29;17:11173. doi: 10.15766/mep_2374-8265.11173 (PMC8319152; doi:10.15766/mep_2374-8265.11173)
Supplement: Supplementary file 1 — Mock SOE Faculty Tip Sheet.pdfPart 1 Slide Presentation.pptxPart 2 Script, Stem, Questions & Evaluation.docxFacilitator Guide.docxFaculty Workshop Evaluation.docxFaculty Preintervention Survey.docxFaculty Postintervention Survey.docxResident Preintervention Survey.docxResident Postintervention Survey.docx [file mep_2374-8265.11173-s001.zip › F. Faculty Preintervention Survey.docx]

**Mock SOE Faculty Pre-Intervention**

In preparation of the upcoming mock Standardized Oral Examination(SOE) Faculty Development session, we are surveying the faculty to better understand your views and practices during administration of mock SOEs. Please complete the following survey to help us tailor the educational session to meet your needs.

Section 1: Demographics

How many years since you have taken the oral boards/APPLIED examination?

- <1 year
- 1-2 years
- 3-5 years
- 6-10 years
- >10 years
- I didn't take the Oral Boards

How many years have you been administering mock SOE?

- <1 year
- 1-2 years
- 3-5 years
- 6-10 years
- >10 years
- Have not administered a mock SOE before

Have you given at least 1 mock SOE in the last 12 months?

- Yes
- No
- I don't remember
- N/A

Section 2: Understanding

Have you ever visited the section of the ABA website relevant to the APPLIED exams?

- Yes
- No
- I don't remember
- I reviewed other information/education about how to conduct a mock oral exam

*Display This Question:*

*If Section 2: Understanding Have you ever visited the section of the ABA website relevant to the App... = Yes*

Have you watched the videos provided by the ABA depicting good/bad candidate performances?

- Yes
- No
- I don't remember

  How familiar are you with the format of the current ABA Standardized Oral Exam?

- Extremely familiar
- Very familiar
- Moderately familiar
- Slightly familiar
- Not familiar at all

Section 3: Preparation

How do you prepare when you will be giving a mock SOE? (Check all that apply)

- Read the question stem
- Look up clinical information when I am unsure of the clinical content
- Create some of my own questions
- Plan the flow of questioning
- I don't prepare
- Other, please specify ________________________________________________

Section 4: Exam Delivery

Do you ask every question that’s provided to you?

- Always
- Most of the time
- About half the time
- Sometimes
- Never

How often do you improvise or add your own questions?

- Always
- Most of the time
- About half the time
- Sometimes
- Never

Section 5: Feedback

How often do you provide feedback on the following?

|  | Always | Most of the time | About half the time | Sometimes | Never |
| --- | --- | --- | --- | --- | --- |
| Answering style |  |  |  |  |  |
| Knowledge of content |  |  |  |  |  |
| Judgment of examinee |  |  |  |  |  |
| Adaptability of examinee |  |  |  |  |  |
| Organization of answers |  |  |  |  |  |

How important do you believe it is for residents to receive feedback in the following areas?

|  | Extremely important | Very important | Moderately important | Slightly important | Not at all important |
| --- | --- | --- | --- | --- | --- |
| Application of Knowledge |  |  |  |  |  |
| Judgment |  |  |  |  |  |
| Adaptability |  |  |  |  |  |
| Organization |  |  |  |  |  |

Section 6: Evaluation

Please indicate your agreement with the following statements.

|  | Strongly agree | Somewhat agree | Neither agree nor disagree | Somewhat disagree | Strongly disagree |
| --- | --- | --- | --- | --- | --- |
| I am very comfortable using the evaluation form provided |  |  |  |  |  |
| I understand the definition of each category the evaluation includes |  |  |  |  |  |
| I think the evaluation helps me provide appropriate feedback to residents |  |  |  |  |  |

Section 7: General Comments

What do you think you do well in conducting a mock SOE?

________________________________________________________________

________________________________________________________________

What do you think you could do better in conducting a mock SOE?

________________________________________________________________

______________________________________________________________
