# Supplementary material for: American Board of Anesthesiology Mock Standardized Oral Examination Faculty Development Workshop
Source: MedEdPORTAL. 2021 Jul 29;17:11173. doi: 10.15766/mep_2374-8265.11173 (PMC8319152; doi:10.15766/mep_2374-8265.11173)
Supplement: Supplementary file 1 — Mock SOE Faculty Tip Sheet.pdfPart 1 Slide Presentation.pptxPart 2 Script, Stem, Questions & Evaluation.docxFacilitator Guide.docxFaculty Workshop Evaluation.docxFaculty Preintervention Survey.docxFaculty Postintervention Survey.docxResident Preintervention Survey.docxResident Postintervention Survey.docx [file mep_2374-8265.11173-s001.zip › G. Faculty Postintervention Survey.docx]

**Mock SOE Faculty Post-Intervention**

As a follow-up to our Mock Oral Faculty Development initiative, we are surveying faculty to determine any changes in attitude or views of our mock oral process.

Did you administer a mock oral on (DATE)? (If you did not, you do not need to complete this survey.)

- Yes
- No

*Skip To: End of Survey If Did you administer a mock oral on (DATE)? (If you did not, you do not need to complete this surv... = No*

How did you participate in the Mock Oral Faculty Development Initiative? (Check all the apply)

- I attended the session on (DATE)
- I reviewed the Mock Oral Tip sheet
- I did not participate

Did you try anything different with the mock oral you administered today?

- Yes
- No

*Display This Question:*

*If Did you try anything different with the mock oral you administered today? = No*

Why not?

________________________________________________________________

________________________________________________________________

________________________________________________________________

________________________________________________________________

________________________________________________________________

*Display This Question:*

*If Did you try anything different with the mock oral you administered today? = Yes*

Please describe what you did differently.

________________________________________________________________

________________________________________________________________

________________________________________________________________

________________________________________________________________

________________________________________________________________

In which areas did you provide feedback to the resident during the mock oral you administered today?

- Answering style
- Knowledge of content
- Application of knowledge
- Judgment of examinee
- Adaptability of examinee
- Organization of answers

Please indicate your agreement with the following statements.

|  | Strongly agree | Somewhat agree | Neither agree nor disagree | Somewhat disagree | Strongly disagree |
| --- | --- | --- | --- | --- | --- |
| I am very comfortable using the evaluation form provided |  |  |  |  |  |
| I understand the definition of each category the evaluation includes |  |  |  |  |  |
| I think the evaluation helps me provide appropriate feedback to residents |  |  |  |  |  |

General Comments

________________________________________________________________

________________________________________________________________

________________________________________________________________

________________________________________________________________

________________________________________________________________
