# Supplementary material for: American Board of Anesthesiology Mock Standardized Oral Examination Faculty Development Workshop
Source: MedEdPORTAL. 2021 Jul 29;17:11173. doi: 10.15766/mep_2374-8265.11173 (PMC8319152; doi:10.15766/mep_2374-8265.11173)
Supplement: Supplementary file 1 — Mock SOE Faculty Tip Sheet.pdfPart 1 Slide Presentation.pptxPart 2 Script, Stem, Questions & Evaluation.docxFacilitator Guide.docxFaculty Workshop Evaluation.docxFaculty Preintervention Survey.docxFaculty Postintervention Survey.docxResident Preintervention Survey.docxResident Postintervention Survey.docx [file mep_2374-8265.11173-s001.zip › I. Resident Postintervention Survey.docx]

**Mock SOE Resident Post-Intervention**

Mock orals are an important part of residency training and preparation for board examinations. We implemented a new initiative to improve residents' experiences with mock orals. The purpose of this survey is to assess whether or not this intervention impacted your mock oral experience. Your responses will be de-identified and only disclosed to faculty involved with this project in aggregate.

Your responses will help us improve the process in the future.
 
Thank you!

Faculty:
*(Information will be used only to pair faculty ratings after they participate in a professional development activity to improve their performance. Responses to this survey will only be disclosed to faculty in aggregate, thus your responses will be anonymous.  If you are uncomfortable with providing the faculty name, please leave blank.)*

________________________________________________________________

Please indicate your agreement with the following statements in regard to the mock oral you had on (DATE):

|  | Strongly agree | Somewhat agree | Neither agree nor disagree | Somewhat disagree | Disagree | N/A |
| --- | --- | --- | --- | --- | --- | --- |
| I felt I performed well during this mock oral exam |  |  |  |  |  |  |
| I received helpful feedback about my performance |  |  |  |  |  |  |
| I think this mock oral exam was helpful in preparing me for my board exam |  |  |  |  |  |  |
| This mock oral exam was similar to other mock oral exams that I have participated in |  |  |  |  |  |  |

Please indicate the areas in which your received feedback during your mock oral: (Check all that apply)

- Application of knowledge
- Organization
- Adaptability
- Judgment

Did you notice something different about this mock oral exam from previous exams you have taken?

- Yes
- Maybe
- No

*Display This Question:*

*If Did you notice something different about this mock oral exam from previous exams you have taken? != No*

Please describe what was different about this mock oral exam.

________________________________________________________________

________________________________________________________________

________________________________________________________________

________________________________________________________________

________________________________________________________________
