# Supplementary material for: American Board of Anesthesiology Mock Standardized Oral Examination Faculty Development Workshop
Source: MedEdPORTAL. 2021 Jul 29;17:11173. doi: 10.15766/mep_2374-8265.11173 (PMC8319152; doi:10.15766/mep_2374-8265.11173)
Supplement: Supplementary file 1 — Mock SOE Faculty Tip Sheet.pdfPart 1 Slide Presentation.pptxPart 2 Script, Stem, Questions & Evaluation.docxFacilitator Guide.docxFaculty Workshop Evaluation.docxFaculty Preintervention Survey.docxFaculty Postintervention Survey.docxResident Preintervention Survey.docxResident Postintervention Survey.docx [file mep_2374-8265.11173-s001.zip › Educational_Summary_Report_11173.pdf]

MedEdPORTAL® The AAMC Journal of Teaching and Learning Resources

Original Publication Open Access

# American Board of Anesthesiology Mock Standardized Oral Examination Faculty Development Workshop

Lauryn R. Rochlen, MD*, Derek T. Woodrum, MD, Lara Zisblatt, EdD, MA, PMME

*Corresponding author: rochlenl@med.umich.edu

## Abstract

**Introduction:** Preparation for oral board examination is an important part of residency training. Anesthesiology programs provide mock oral exams for their trainees, but often, faculty have little guidance on the conduct of these exams. We describe a faculty development workshop for anesthesiology faculty to enhance their familiarity with the American Board of Anesthesiology Standardized Oral Examination (SOE). **Methods:** We created a faculty development workshop to administer to a live audience. The session consisted of didactic and practical components. A one-page tip sheet was also included to distribute to all faculty administering mock SOEs, for review and reference prior to administering an exam. Faculty and residents were surveyed before and after the session. **Results:** Eleven faculty participated in the live session. Eighty-two percent of faculty (nine of 11) committed to making a change in the way they delivered mock SOE as a result of attending the session. Fifty-eight percent of faculty (32 of 55) who responded to the postintervention survey reported that they used the tip sheet prior to administering a subsequent mock SOE. Residents described improvement in the clarity and organization of feedback following the intervention. **Discussion:** Faculty members play a vital role in preparing residents for board certification. It is therefore important that faculty are appropriately oriented to the goals and conduct of the mock SOE. After taking this workshop, faculty members will be more likely to adapt their examiner style to focus on the ABA-defined examinee attributes and to provide feedback after the mock SOE.

**Keywords**
ABA Certification, Oral Board Preparation, Faculty Development, Standardized Oral Examination, Licensure, Certification, Anesthesiology

## Educational Objectives

By the end of this activity, learners will be able to:

1. Describe the format of the American Board of Anesthesiology APPLIED Standardized Oral Examination.
2. Administer exams by emphasizing decision-making, organization, adaptability, and application of knowledge.
3. Deliver appropriate feedback to the resident at the end of the examination.

## Introduction

Board certification by the American Board of Anesthesiology (ABA) allows an anesthesiologist to demonstrate competency and is often required for employment opportunities.[1] The ABA continues to maintain a three-part certification process, consisting of two written examinations and a multicomponent oral examination known as the APPLIED examination. The latter is taken after completion of residency (or graduate medical training) and consists of two parts: the Standardized Oral Examination (SOE) and the Objective Structured Clinical Examination.

Anesthesiology training programs must obtain a >70% board certification rate to maintain their accreditation through the Accreditation Council for Graduate Medicine Education.[2] In a recent survey published by Isaak and colleagues, 91% of the responding anesthesiology training programs agreed that the training program was responsible for preparing their trainees for the certification examination process.[2] In this same survey, 100% of responding programs indicated they provided preparation by administering mock SOEs (mSOEs). What is not currently understood is how anesthesiology training programs conduct these mock exams in terms of frequency, resident preparation, assessment, and feedback.

**Citation:**
Rochlen LR, Woodrum DT, Zisblatt L. American Board of Anesthesiology mock Standardized Oral Examination faculty development workshop. *MedEdPORTAL*. 2021;17:11173.
https://doi.org/10.15766/mep_2374-8265.11173

Copyright © 2021 Rochlen et al. This is an open-access publication distributed under the terms of the Creative Commons Attribution license. 

Development of an mSOE program at one institution is detailed in an article by Schubert and colleagues published in 1999.[3] Anesthesiology faculty in that study attended an annual in-service session about practice oral exams. Each faculty examiner would also receive a briefing packet a few days prior to exam administration. In their follow-up to this initial study, their team was able to show consistent interrater reliability of mSOE evaluations as well as validity for their assessment tool.[4]

Our institution is not alone in having no formal education or training process for the faculty who participate in the mSOE program, nor do we have a method to assess how faculty administer the mSOE and provide resident feedback. We conducted a needs assessment survey of 148 faculty as part of our Faculty Preintervention Survey and found that 64% of faculty (14 of 22) who answered the survey had not reviewed the relevant ABA information about the SOE and that 23% (five of 22) considered themselves only slightly familiar or not familiar at all with the format of the SOE. At our institution, we have board examiners trained by the ABA in exam administration. As a result, we have been able to create a workshop to share this experience with faculty colleagues who administer mSOEs. Therefore, the goal of this *MedEdPORTAL* resource is to detail a workshop created to enhance anesthesiology faculty understanding of the ABA APPLIED SOE and help standardize the feedback given to trainees about their performance on the mSOE.

All information included within this publication concerning the APPLIED SOE is in line with information publicly available from the ABA. This faculty development session and associated materials are purely intended to be used for educational purposes. The evaluation form included is widely available and used at many institutions and is referenced in the Schubert and colleagues article.[3] The responsibility for the content of the submission lies with the authors and not with *MedEdPORTAL* or the ABA.

There are other *MedEdPORTAL* publications that provide resources for faculty development workshops relating to board prepartion.[5-7] Our resource is novel in that it is focused on the topic of faculty preparation for administering mSOEs for anesthesiology specialty certification and will therefore be helpful to other anesthesiology residency programs wanting training that is more specialty focused.

## Methods

All clinical faculty members of the Department of Anesthesiology at the University of Michigan are expected to participate in the administration of mSOEs. We conduct five mSOEs each year. To train the faculty on how to properly administer, assess, and provide feedback, an evening workshop was scheduled. All faculty participating in the mSOEs were invited to attend the workshop.

Authors Lauryn R. Rochlen and Derek T. Woodrum serve as APPLIED board examiners for the ABA. This faculty development workshop was developed in order to share their expertise with other faculty members in the department. The goal of the workshop was to improve faculty awareness of the SOE structure, increase understanding of the attributes assessed on the SOE, improve the quality and consistency of the administered exams, and enhance the feedback process.

In addition, a tip sheet that highlighted the main points of the workshop was distributed to all faculty members to allow for a broader reach of the education (Appendix A: Mock SOE Faculty Tip Sheet).

### Faculty Workshop

This workshop was intended for anesthesiology faculty members involved in administering mSOEs to anesthesiology residents or recent graduates preparing for their APPLIED examination. The workshop consisted of two parts, lasted 1 hour, and took place in a conference room with access to a computer and projector.

The workshop was set up as follows:

- Part 1. Didactic session (30 minutes; Appendix B: Part 1 Slide Presentation): The didactic session covered the basic components of the SOE. We used our established SOE evaluation form, which was developed based on the grading of SOE by the ABA and had been used for many years in our department, to structure the educational material. Most faculty were very familiar with the evaluation form (Appendix C: Part 2 Script, Stem, Questions & Evaluation).
- Part 2. Role-play with two faculty facilitators (30 minutes; Appendix C: Part 2 Script, Stem, Questions & Evaluation).

The Facilitator Guide (Appendix D) contained the details for running this workshop. Appendix E is the evaluation for the workshop.

### Outcomes Assessment

*Evaluation and survey development:* Since this activity aimed at the development of faculty, we used the outcomes model developed by Moore, Green, and Gallis[8] to assess the effectiveness of the intervention as that was the model most commonly used for continuing medical education. Using this

Copyright © 2021 Rochlen et al. This is an open-access publication distributed under the terms of the Creative Commons Attribution license.

model, we developed tools that assessed our curriculum's ability to achieve each level of outcome. These included assessing participation, satisfaction, self-assessed knowledge and competency, self-reported changes in practice, and changes in practice reported by residents. Each survey was developed by an educational specialist (author Lara Zisblatt) with a doctorate in medical education and a certificate in program evaluation. The surveys were then reviewed by the other authors to assess content validity and tested by two respondents. Using cognitive interviewing, recommendations for changes were gathered, and changes were made to the survey.

*Faculty surveys (Appendix F: Faculty Preintervention Survey and Appendix G: Faculty Postintervention Survey):* All faculty members were sent an email inviting them to the mSOE faculty development workshop. The email included a link to a brief Faculty Preintervention Survey intended to collect baseline data prior to the workshop. As a result, the preintervention survey was delivered to all participants before the workshop regardless of their intent to attend the workshop. The survey assessed demographics, understanding of the SOE, faculty preparation for an mSOE, methods of delivery of the SOE, and type(s) of feedback provided to the resident following the mSOE. After each mSOE, every faculty who conducted an SOE received the Faculty Postintervention Survey asking about their experiences, whether they attended the workshop and/or reviewed the tip sheet, and whether or not they tried a different technique in conducting the mSOE.

*Resident surveys (Appendix H: Resident Preintervention Survey and Appendix I: Resident Postintervention Survey):* Before the workshop, the Resident Preintervention Survey was sent to all residents asking about their experience with mSOEs in which they had participated that day. After their mSOE in the post–faculty development workshop era, residents were asked to complete the Resident Postintervention Survey.

### Statistical Analysis

Comparison of proportions was calculated using MEDCALC (MedCalc Software, 2019) to determine if participation in the faculty development initiative was correlated to changes in competence, attitudes, or behavior. Because this research met education exemption standards (established education settings, normal education practice, without impact on students or teachers, and without personal information), the study did not require full review by the institutional review board.

## Results

To evaluate the session, we followed the Moore, Green, and Gallis expanded outcomes framework for assessing continuing medical education activities.[8] Per this model, we assessed participation, satisfaction, changes in knowledge, self-reported changes in performance, and observed changes in performance as reported by residents receiving the SOE.

### Participation

Eleven out of 148 faculty members attended the workshop, and 55 Faculty Postintervention Surveys were completed. Results showed that 58% of faculty (32 of 55) who completed surveys reviewed the Mock SOE Faculty Tip Sheet prior to administering an mSOE.

### Satisfaction

Of the faculty who attended the workshop, 100% (11 of 11) said they were satisfied with the session, with 82% (nine of 11) reporting they were extremely satisfied with it. In addition, over 90% of participants felt that all objectives were fully met.

### Faculty Commitment to Change Practice

Eighty-two percent of faculty (nine of 11) committed to making a change in the way they delivered mSOEs as a result of attending the session. For example, five participants discussed not focusing on content as much, and three discussed putting more focus on the ABA attributes under assessment. In addition, three participants discussed the importance of leaving more time for feedback, and two discussed using more probing questions.

### Self-Reported Change in Competence and Attitudes

For self-reported changes in competence and attitudes, we analyzed the data in two ways. We looked at the percentage of faculty who strongly agreed with statements designed to assess their competence and attitudes toward the mSOE evaluation sheet. First, we looked at the difference in the Faculty Preintervention Survey results ($n = 22$) versus the Faculty Postintervention Survey results ($n = 55$). In all categories, a higher percentage of faculty strongly agreed with the statements more favorable to using the evaluation sheet. Figure 1 shows the statements and the difference in those who strongly agreed preintervention versus postintervention.

In addition, we looked at only the postintervention data and saw that faculty who participated in the initiative (attended the workshop or reviewed the tip sheet) were more likely to strongly agree with statements about their perceived competence and attitudes toward using the evaluation sheet to conduct the mSOE. Figure 2 shows the specific results.

Copyright © 2021 Rochlen et al. This is an open-access publication distributed under the terms of the Creative Commons Attribution license.

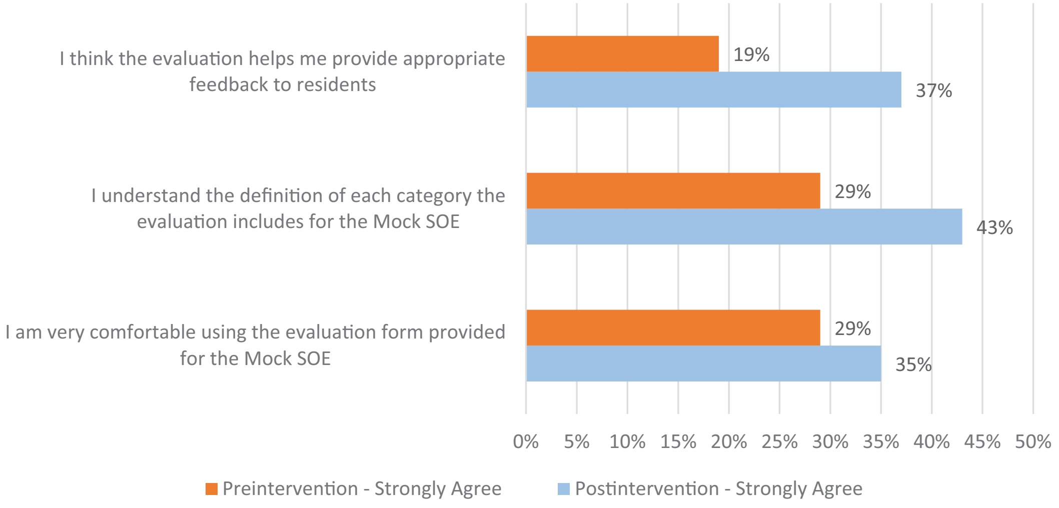


**Figure 1.** Percentage of faculty who self-reported changes in competence and attitudes postintervention. Abbreviation: SOE, Standardized Oral Examination.

Self-Reported Changes in Practice

All faculty who were eligible to administer mSOEs, whether or not they attended the workshop or reviewed the tip sheet, were sent the Faculty Preintervention Survey and Faculty Postintervention Survey. The email to the faculty asked only those who had ever administered an mSOE to complete the preintervention survey, and only those who completed an mSOE after the date of the workshop to complete the postintervention survey. We included a question in the survey to ensure that those who did not complete an mSOE would not submit results. In addition, we asked people who had already completed the postsurvey to not complete the survey again even if they administered an mSOE more than once during the 6-month period after the intervention.

Over the next 6 months, we held three mSOE sessions, for a total of 142 exams. The Faculty Postintervention Survey was sent out right after the administration of mSOEs for the next 6 months. In that time, three mSOEs were conducted, with the Faculty Postintervention Survey being sent to all faculty after each mSOE. Fifty-five surveys were completed. Of the 55 respondents, 10 had attended the workshop, while 32 reviewed the Mock SOE Faculty Tip Sheet only (those who attended the workshop may have also reviewed the tip sheet) and 13 did not participate in any part of the intervention. When asked if they tried anything different with the mSOE they administered, 50% (21 of 42) of those who completed at least one part of the intervention reported trying something different, while only 8% (one of 12) of faculty who did not participate reported trying something different (not all faculty

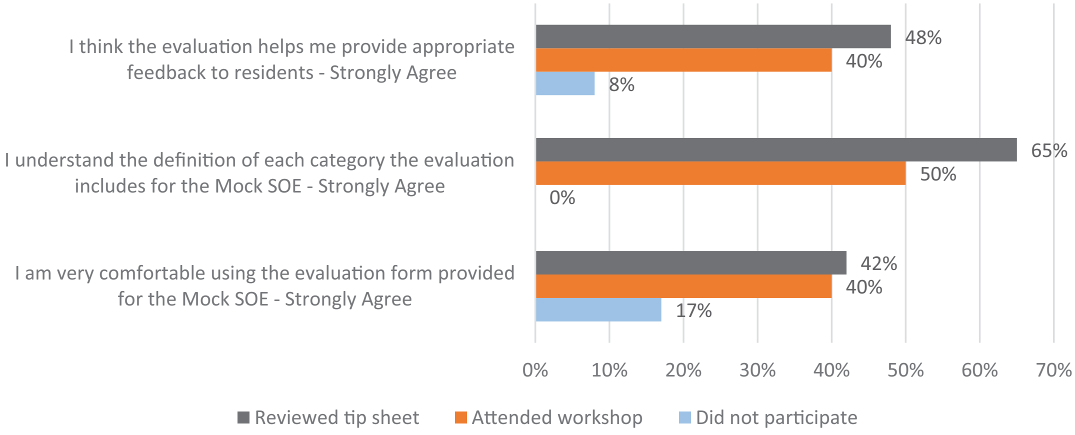


**Figure 2.** Percentage of faculty who strongly agreed with the reported statements based on their participation in the mock SOE initiative. Abbreviation: SOE, Standardized Oral Examination.

Copyright © 2021 Rochlen et al. This is an open-access publication distributed under the terms of the Creative Commons Attribution license.

completed this question). A chi-square test of independence was performed to examine the relation between participation in the interventions and likelihood to make changes in practice. The relation between these variables was significant ($\chi^2 = 7.4$, $p = .0065$). Faculty who participated either by attending the workshop or reviewing the tip sheet were more likely to self-report changes in practice (Figure 3).

Common examiner changes reported included the following:

- Asked more questions to determine thought process, asked "why" (six respondents).
- Fewer cues, less teaching during mock oral exam (four respondents).
- Made feedback to residents more about the attributes (three respondents).
- No facial feedback (three respondents).

Some direct quotes included the following:

- "Didn't read verbatim from the sheet."
- "Using the common candidate problems and solutions recommendations helped to identify the resident's pattern of answering questions. The suggested solutions help approach different answering styles. Review of the tips allowed me to adapt my testing quicker and more efficiently than before."
- "Did not ask specific questions about 'what potassium would you proceed with' but instead created a scenario where someone 'accidentally pushed succinylcholine' in a renal failure patient and the patient developed EKG changes. Then asked them how they would lower potassium :)"
- "With one word answers—yes or no—I asked them why or why not to encourage them to elaborate. If they wanted further tests, I asked them how it would change their management. And when they struggled, I said, 'let's move on.' I found the tips quite helpful."

Resident-Reported Changes in Practice

While there were no differences found in most of the answers given between the Resident Preintervention Survey versus the Resident Postintervention Survey, 43% of residents (31 of 72) answered either yes or maybe to the question asking whether they noticed a difference in how the exam was administered by the faculty after the intervention. There were 25 comments in total. We analyzed the comments to determine categories and themes. Comments assessed either the exam itself (three) or the examiner (20). (In the two additional comments that did not fit this dichotomy, residents misinterpreted the question and answered how they felt they did on the mock oral.) One category concerned the focus of the comment. The comment was either focused on the content of the exam (six comments), the style of the exam (15), or the logistics of the exam (two). The themes related to the content of the exam were about complexity of the patient or difficulty of the exam; for instance, "The question stem had a less complex patient, in my opinion." The themes related to the style of the exam spoke about examiners and discussed receiving better feedback or more focus on how the exam was given. These themes included comments that mentioned examiners doing the following:

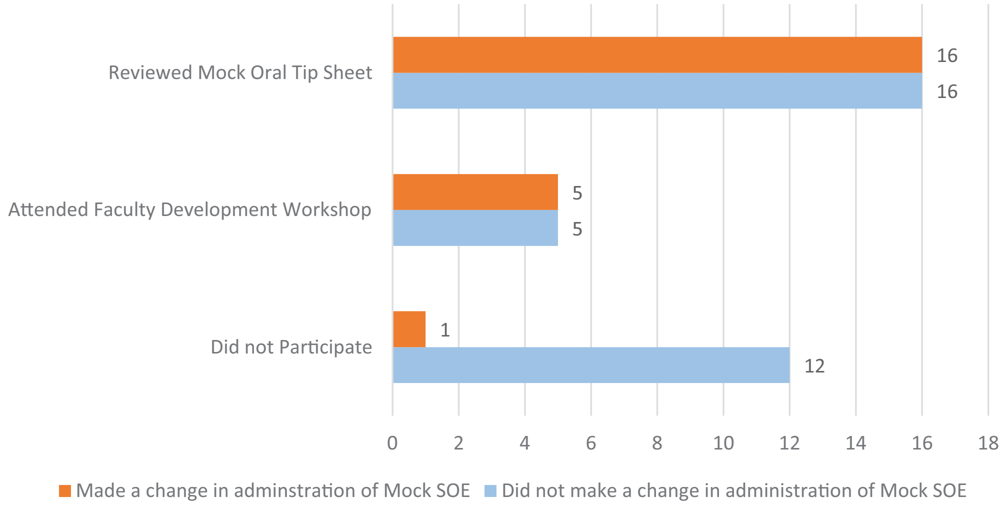


**Figure 3.** Number of faculty who answered yes or no to questions about participating in the mock SOE initiative and making changes to their practice. Abbreviation: SOE, Standardized Oral Examination.

Copyright © 2021 Rochlen et al. This is an open-access publication distributed under the terms of the Creative Commons Attribution license.

- Asking why more often:
  - "More push-back on alternatives mentioned in the care and having me justify their choices."
  - "Pushed a little more on my confidence in answers."
- Focusing on less prompting:
  - "Less prompting, which helps for the oral exam after residency."
  - "Examiner would let me talk until I stopped and did not give cues if I was saying correct info or on correct path."
- Providing better feedback:
  - "Great explanation about how real oral exam will go, specific examples from my answers where I can improve."
  - "More focused feedback on stylistic elements (organizing thoughts, not rambling, etc.)."

Figure 4 shows a diagram of the categories and subcategories from this qualitative analysis of the comments.

## Discussion

This faculty development workshop was created to enhance anesthesiology faculty understanding of the ABA SOE and to assist them when preparing to be mSOE examiners. While not every faculty member was able to attend the in-person development workshop, the one-page Mock SOE Faculty Tip Sheet summarized the key points of the discussion and was sent to all faculty participating in the mSOE program at our institution.

Results from the multiple surveys of faculty and residents revealed that this workshop and the tip sheet met our objectives. All of the faculty who attended the live session were satisfied with the content and delivery of the material. The majority of them committed to making a change in how they would conduct their mSOEs as a direct result of their participation. Some of these faculty stated they would focus less on content and more on the four major candidate attributes. The faculty also said they would leave more time for feedback at the end of the mSOE. As only a small proportion of the faculty was able to attend the live session, we plan to hold additional sessions in the future to engage those who were unable to attend.

One of the overall goals of creating this workshop and the tip sheet was to help faculty prepare to administer a meaningful mSOE for the residents. In the preintervention survey, we learned that there were faculty who were hesitant to participate in the mSOE program due to anxiety around clinical knowledge. The workshop and tip sheet should have improved their comfort with the process, as well as with what was expected of them in the process. We continued to reiterate that the mSOE was not the time for didactic teaching.[4]

Based on the prior publications evaluating the mSOE experiences, Schubert and colleagues emphasized that it is not the grading that is important.[3] The focus should be on providing guidance to the trainee on how to improve their approach to answering the question and offering suggestions for improvement. The examiners should be instructed not to teach didactic material. We saw that this was a difficult concept for faculty to understand. One of the objectives of the workshop was to reinforce this concept.

Ultimately, the goal of the workshop was to have a direct impact on the experience of the residents. While faculty might have learned something and committed to change, we were hopeful that the changes would have a positive impact on the residents. Some of the residents did report a difference in how their exams were administered following the intervention. They also noted more clarity and organization surrounding feedback. With repeated sessions for faculty and continued reminders about

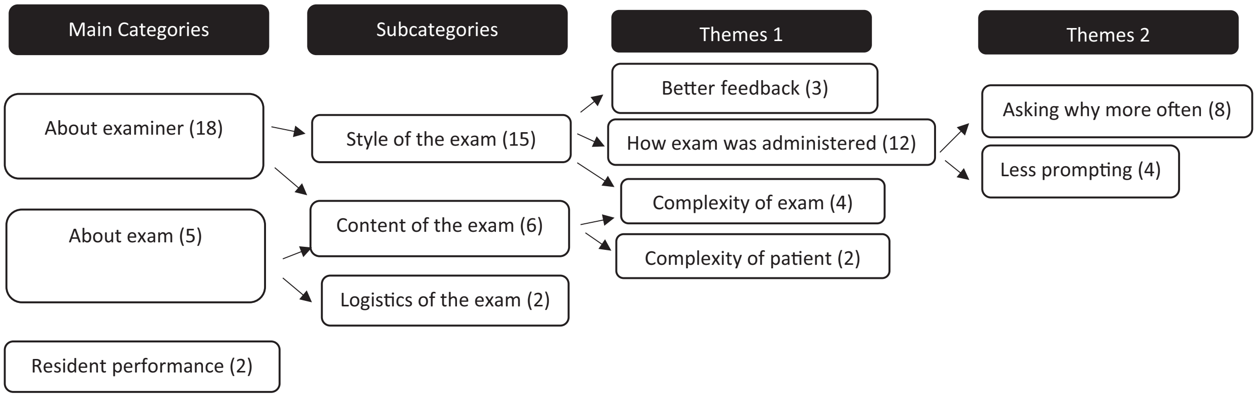


**Figure 4.** Diagram of categories and subcategories from qualitative analysis of comments.

Copyright © 2021 Rochlen et al. This is an open-access publication distributed under the terms of the Creative Commons Attribution license.

the tip sheet, we hope to see a larger effect on the residents with future mSOEs.

As noted in the Introduction, the information contained within this publication is in line with that publicly available from the ABA. There is no proprietary information included. The evaluation form is based on the ABA form and modeled after previously published evaluation forms.[3] We have shared our expertise for educational purposes, with no financial incentives, and have met all ABA guidelines in creating this resource.

Lessons learned include the following:

- The tip sheet extended the education to faculty who were not able to attend the in-person workshop.
- Participants wanted opportunities to practice different techniques for administering the mSOE on each other during the faculty development workshop.
- Common misconceptions faculty had about the mSOE included:
    - Focusing too much on the content and not on the attributes or the delivery of the questions.
    - Asking too many yes-or-no questions or asking pointed questions about medical knowledge that did not pertain to the question stem.
    - Teaching during the mSOE instead of letting the resident go through the whole process and then providing feedback at the end.

A limitation to this project was the small number of faculty who attended the in-person faculty development session. In addition, more faculty completed the postsurvey versus the presurvey. This was due to the fact that faculty had only one opportunity to complete the presurvey before the workshop was held but received the postsurvey after completing each scheduled mock oral after the intervention. Since the surveys were anonymous, there was no way for us to pair the responses, which was a major limitation to preintervention and postintervention comparisons. In addition, there was no way to know how many individual faculty participated in the SOE because of the lack of documentation kept on the examiners. However, most of the data analysis drew conclusions from preintervention and postintervention survey results independently. For example, the self-report of changes in practice or the residents noticing changes in the way the mSOE was administered denoted a positive impact without relying on a comparison to preintervention data. Also, even in instances where comparisons from pre- to postintervention surveys were not statistically significant, the practical implications of faculty rating themselves as having greater competence and more positive attitudes about the mSOE evaluation process suggest there was more faculty buy-in to the process. Our inability to match faculty who participated in the workshop and/or who self-reported using the tip sheet was a result of allowing residents to provide feedback that did not directly name the faculty administering the exam. We believed that if residents were required to name the faculty member, they would have been less likely to provide honest feedback.

Also, there was an overrepresentation of those who attended the session completing the survey, which could have biased the data. In addition, residents could have completed more than one evaluation about exams in the 6-month postintervention period. This might have led to an underrepresentation of changes noticed, since only the first time that residents experienced a change would it have been one to report. There were additional faculty who utilized the Mock SOE Faculty Tip Sheet prior to administering mSOEs, which may have diluted the data. We also collected data after multiple mSOE sessions, which may have resulted in different behaviors based on retention of learning or effects of multiple mSOE administrations.

Passing the SOE is crucial to achieving primary board certification in anesthesiology. With workshops such as this, residency programs can provide guidance to their faculty on best practices for administering mSOEs as well as offering meaningful practice for their trainees.

## Appendices

A. Mock SOE Faculty Tip Sheet.pdf
B. Part 1 Slide Presentation.pptx
C. Part 2 Script, Stem, Questions & Evaluation.docx
D. Facilitator Guide.docx
E. Faculty Workshop Evaluation.docx
F. Faculty Preintervention Survey.docx
G. Faculty Postintervention Survey.docx
H. Resident Preintervention Survey.docx
I. Resident Postintervention Survey.docx

*All appendices are peer reviewed as integral parts of the Original Publication.*

**Lauryn R. Rochlen, MD:** Clinical Associate Professor, Department of Anesthesiology, University of Michigan Medical School; ORCID: https://orcid.org/0000-0003-0273-4799

Copyright © 2021 Rochlen et al. This is an open-access publication distributed under the terms of the Creative Commons Attribution license.

**Derek T. Woodrum, MD:** Clinical Associate Professor, Department of Anesthesiology, University of Michigan Medical School

**Lara Zisblatt, EdD, MA, PMME:** Education Specialist, Department of Anesthesiology, University of Michigan Medical School

**Disclosures**
None to report.

**Funding/Support**
None to report.

**Ethical Approval**
Reported as not applicable.

## References

1. Culley DJ, Sun H, Harman AE, Warner DO. Perceived value of board certification and the Maintenance of Certification in Anesthesiology program (MOCA). *J Clin Anesth*. 2013;25(1): 12-19. https://doi.org/10.1016/j.jclinane.2012.09.001
2. Isaak RS, Chen F, Arora H, Martinelli SM, Zvara DA, Stiegler MP. A descriptive survey of anesthesiology residency simulation programs: how are programs preparing residents for the new American Board of Anesthesiology APPLIED certification examination? *Anesth Analg*. 2017;125(3):991-998. https://doi.org/10.1213/ANE.0000000000002189
3. Schubert A, Tetzlaff JE, Licina M, Mascha E, Smith MP. Organization of a comprehensive anesthesiology oral practice examination program: planning, structure, startup, administration, growth, and evaluation. *J Clin Anesth*. 1999;11(6):504-518. https://doi.org/10.1016/S0952-8180(99)00085-9
4. Schubert A, Tetzlaff JE, Tan M, Ryckman JV, Mascha E. Consistency, inter-rater reliability, and validity of 441 consecutive mock oral examinations in anesthesiology: implications for use as a tool for assessment of residents. *Anesthesiology*. 1999;91(1): 288-298. https://doi.org/10.1097/00000542-199907000-00037
5. Saeed S, Quock R, Lott J, Kashani N, Woodall W. Building resilience for wellness: a faculty development resource. *MedEdPORTAL*. 2017;13:10629. https://doi.org/10.15766/mep_2374-8265.10629
6. Servey J, Wyrick K. Teaching clinical precepting: a faculty development workshop using role-play. *MedEdPORTAL*. 2018;14:10718. https://doi.org/10.15766/mep_2374-8265.10718
7. Loyal J, Porto A, Camenga D. Creating a program for junior faculty professional development: a tool kit. *MedEdPORTAL*. 2018;14:10703. https://doi.org/10.15766/mep_2374-8265.10703
8. Moore DE Jr, Green JS, Gallis HA. Achieving desired results and improved outcomes: integrating planning and assessment throughout learning activities. *J Contin Educ Health Prof*. 2009;29(1):1-15. https://doi.org/10.1002/chp.20001

**Received:** May 1, 2020
**Accepted:** May 17, 2021
**Published:** July 29, 2021

Copyright © 2021 Rochlen et al. This is an open-access publication distributed under the terms of the Creative Commons Attribution license.
